# Supplementary material for: Current Perspectives on Biological Screening of Newly Synthetised Sulfanilamide Schiff Bases as Promising Antibacterial and Antibiofilm Agents
Source: Pharmaceuticals (Basel). 2024 Mar 22;17(4):405. doi: 10.3390/ph17040405 (PMC11053482; doi:10.3390/ph17040405)
Supplement: Supplementary file 1 [file pharmaceuticals-17-00405-s001.zip › pharmaceuticals-2896641-supplementary.pdf]

Supplementary Materials

Current Perspectives on Biological Screening of Newly Synthetised Sulfanilamide Schiff Bases as Promising Antibacterial and Antibiofilm Agents

Maria Coanda, Carmen Limban, Constantin Draghici, Anne-Marie Ciobanu, Georgiana Alexandra Grigore, Marcela Popa, Miruna Stan, Cristina Larion, Speranta Avram, Catalina Mares, Mariana-Catalina Ciornei, Aura Dabu, Ariana Hudita, Bianca Galateanu, Lucia Pintilie and Diana Camelia Nuta

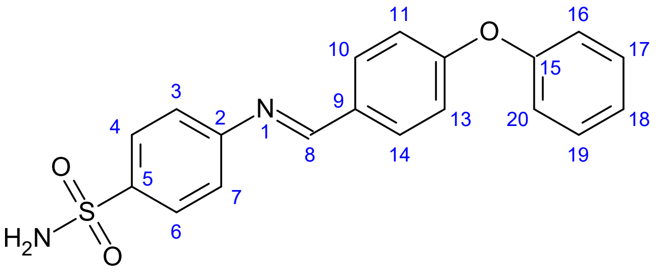

1a

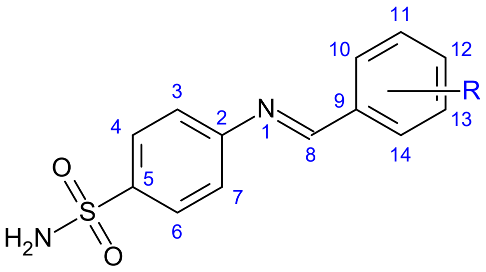

1b-e

*Figure S1. NMR atom numbering in molecules 1a–e. R: 2-Br (**b**); 2,6-diCl (**c**); 3,5-diCl (**d**); 2,3,5-triCl (**e**).*

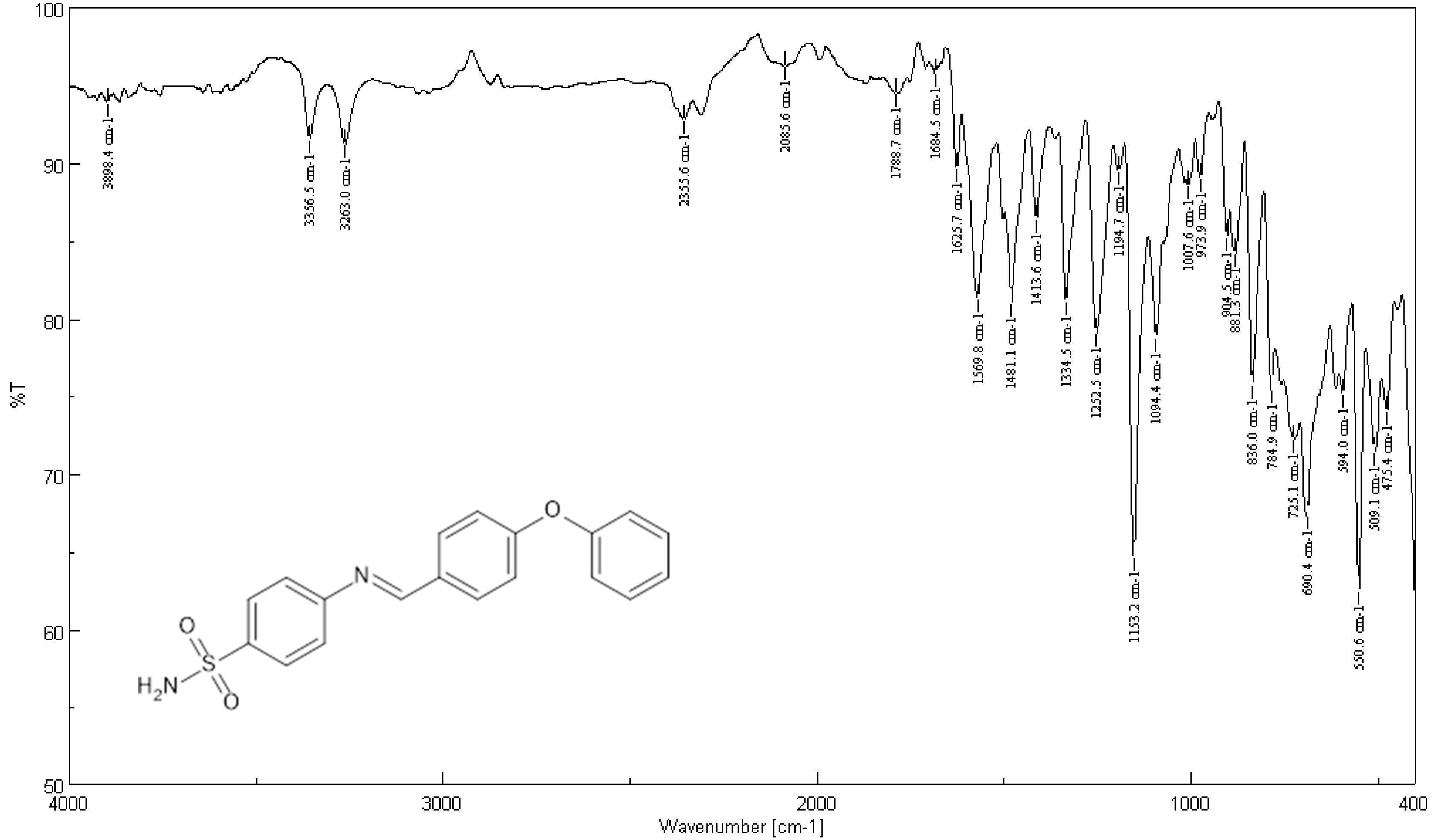

*Figure S2. FT-IR spectrum of **1a**.*

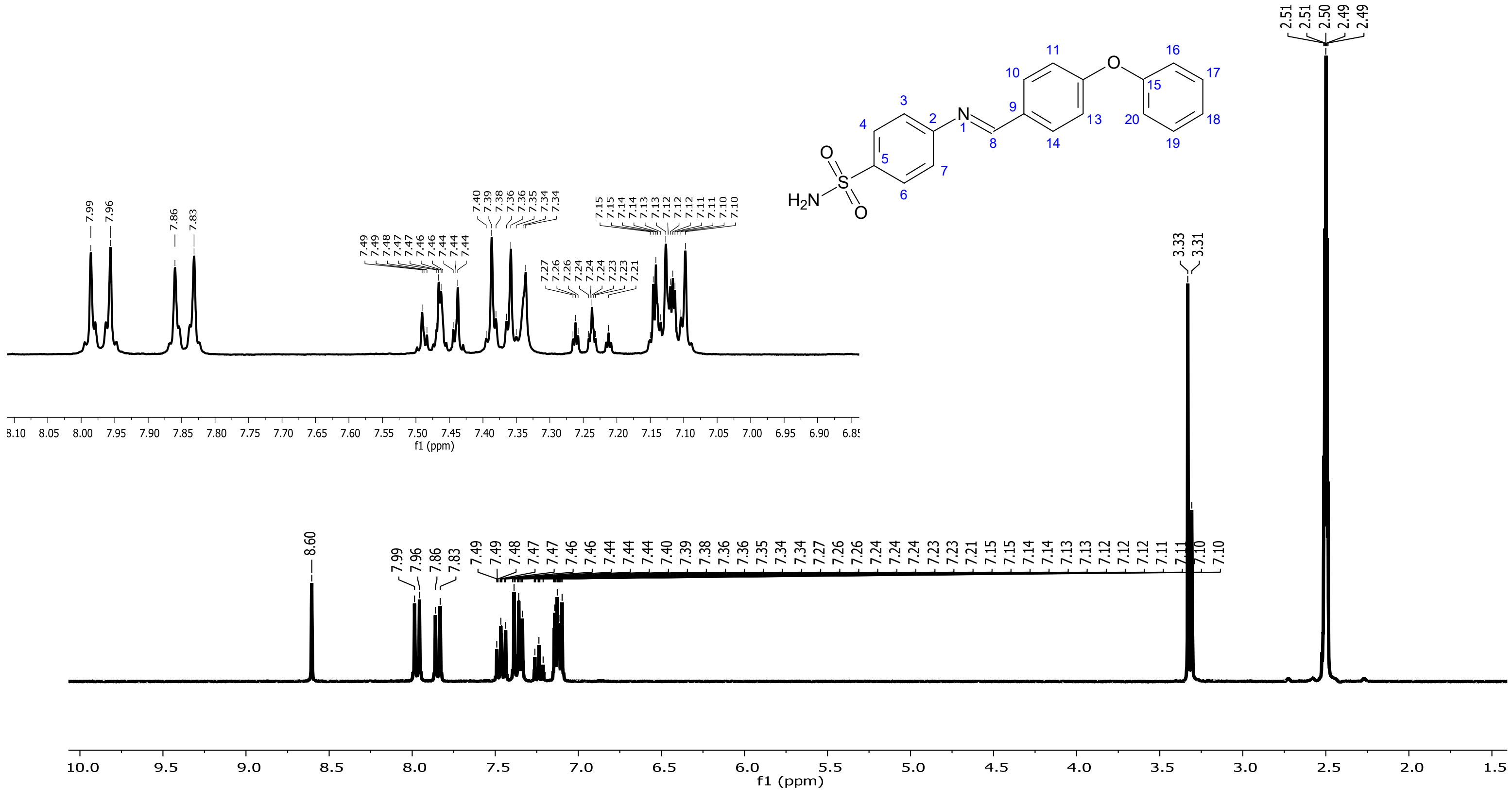

*Figure S3. ^{1}H-NMR spectrum of **1a**.*

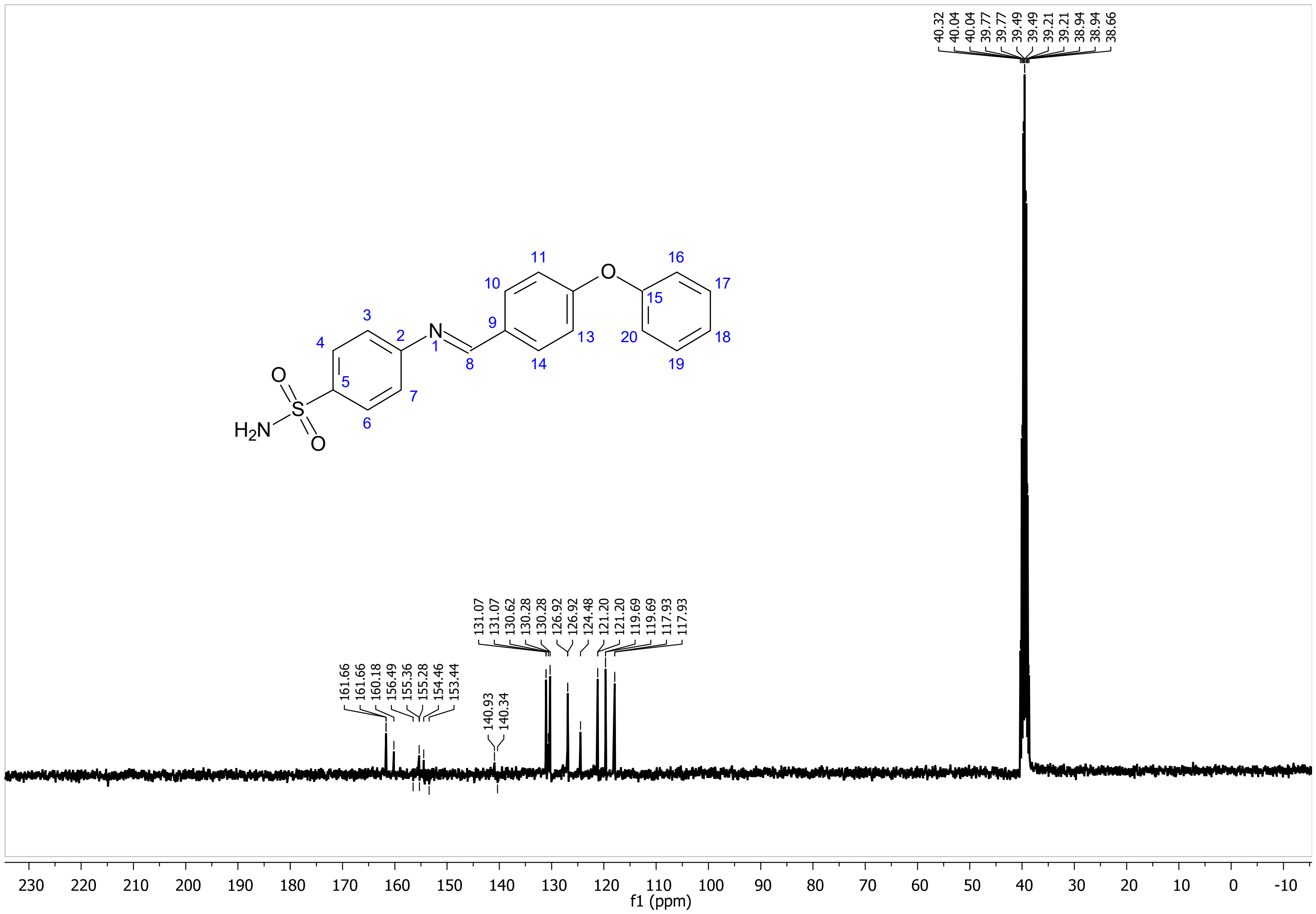

Figure S4. ^{13}C-NMR spectrum of ***1a****.*

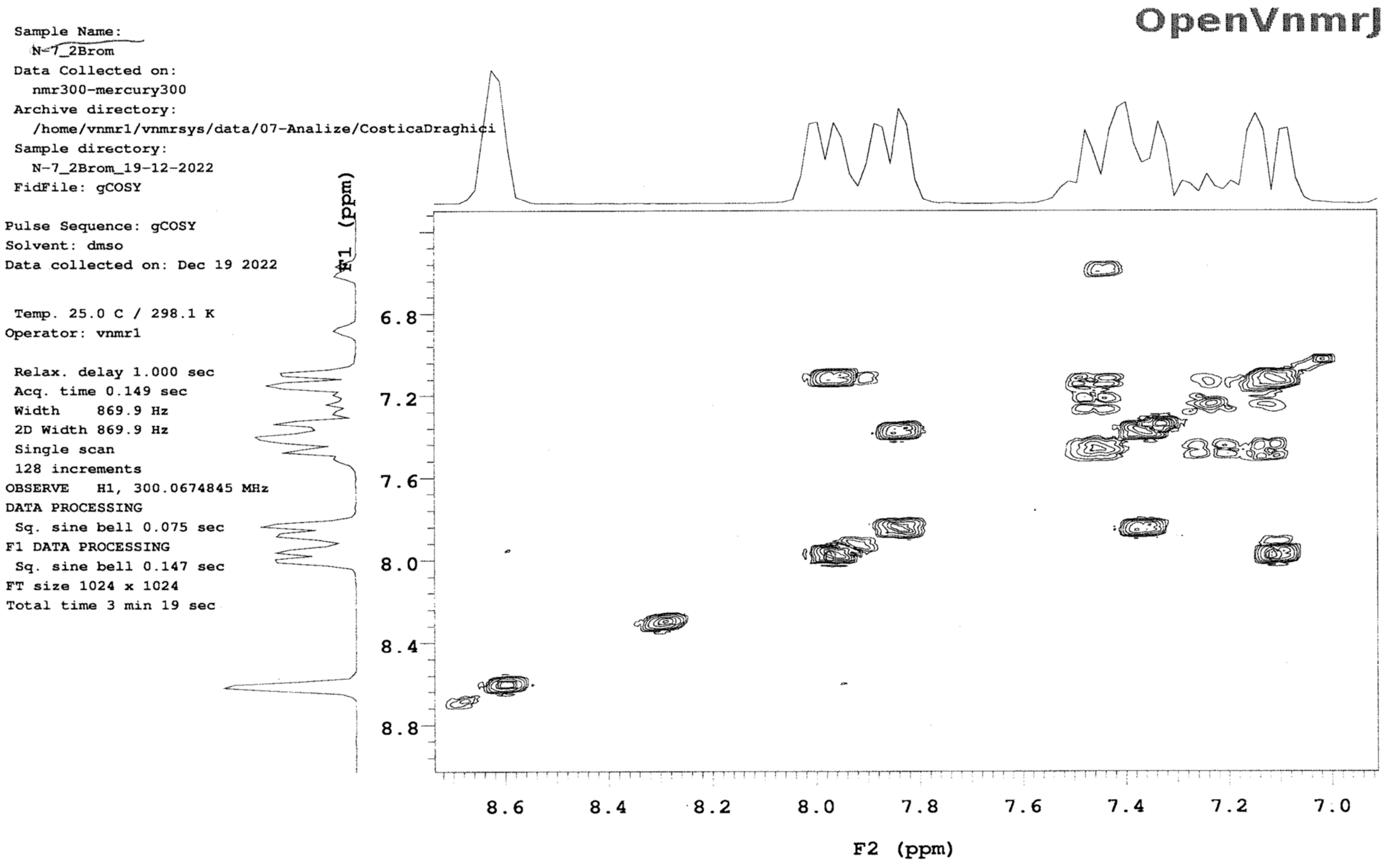

*Figure S5. COSY spectrum (Correlated Spectroscopy) of **1a**.*

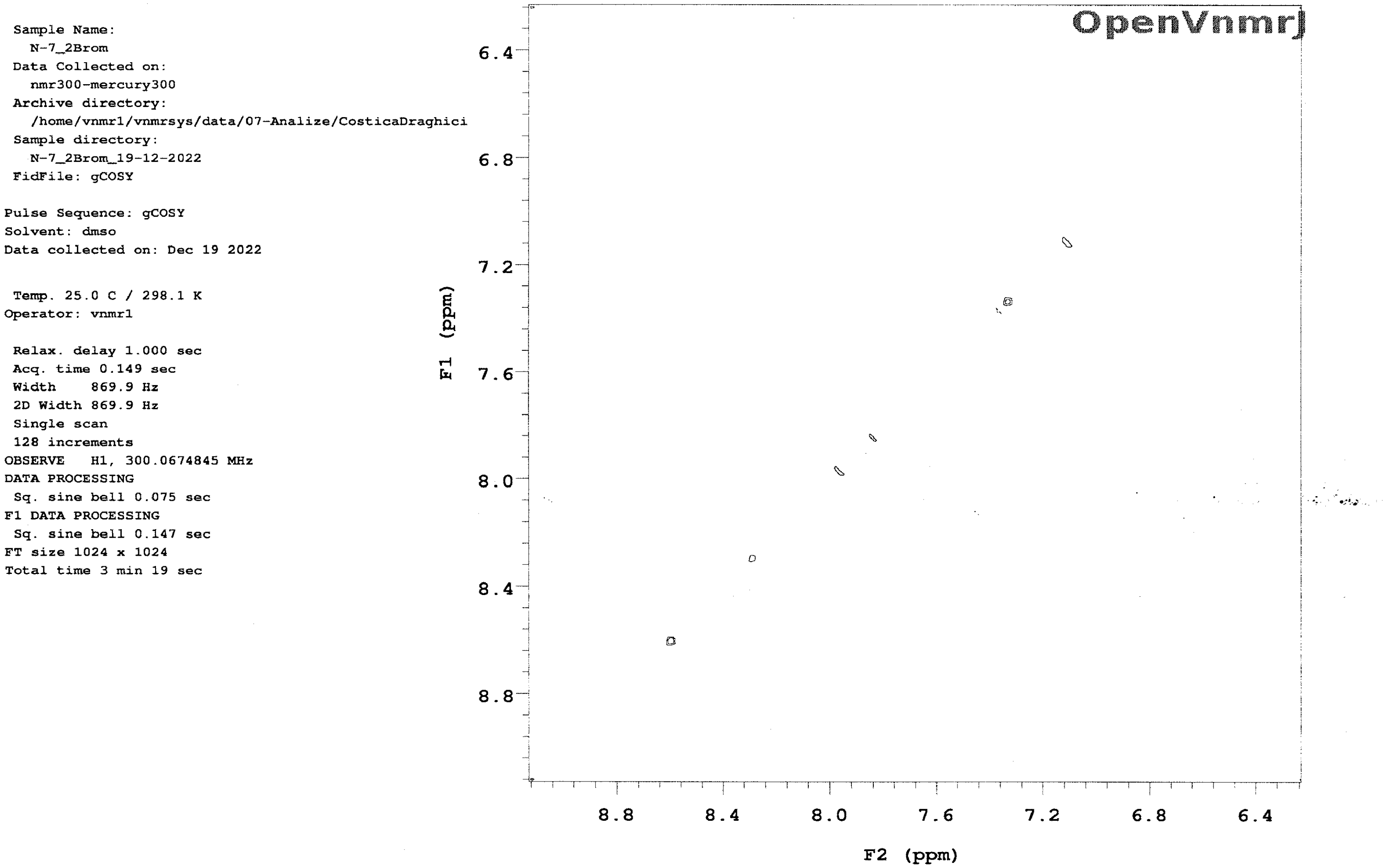

*Figure S6. COSY spectrum (Correlated Spectroscopy) of **1a**.*

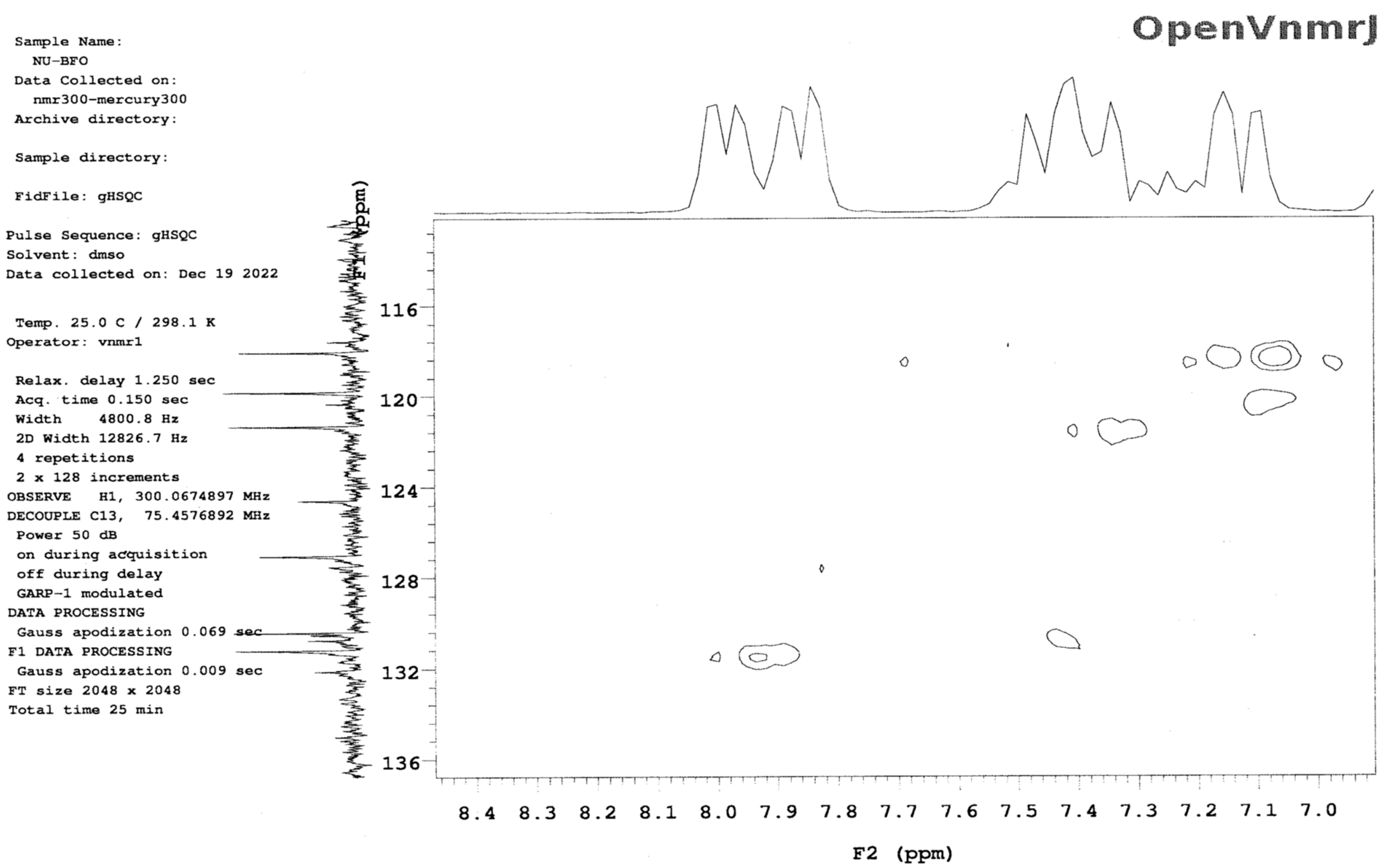

*Figure S7. HSQC spectrum (Heteronuclear Single Quantum Coherence) of **1a**.*

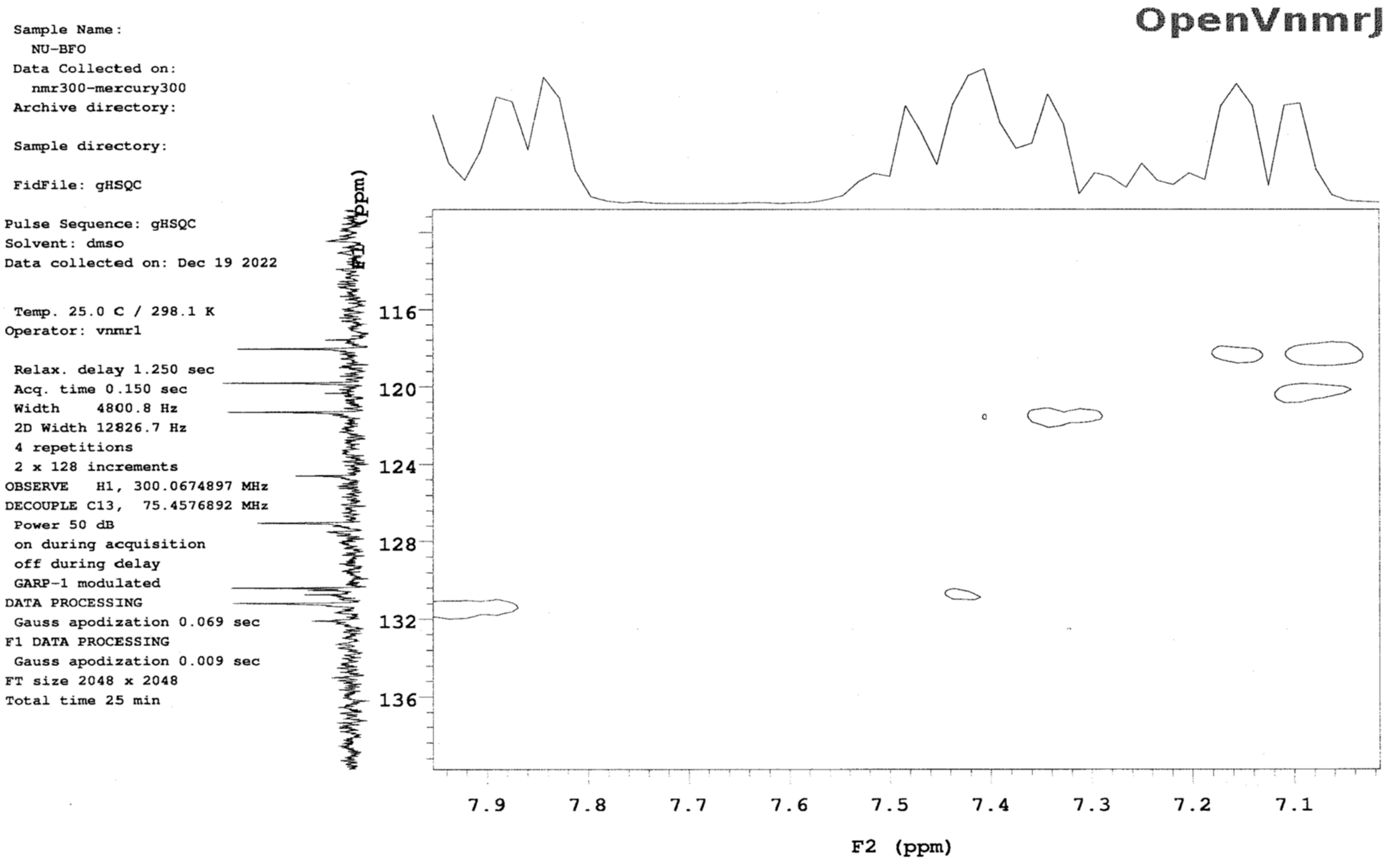

*Figure S8. HSQC spectrum of **1a**.*

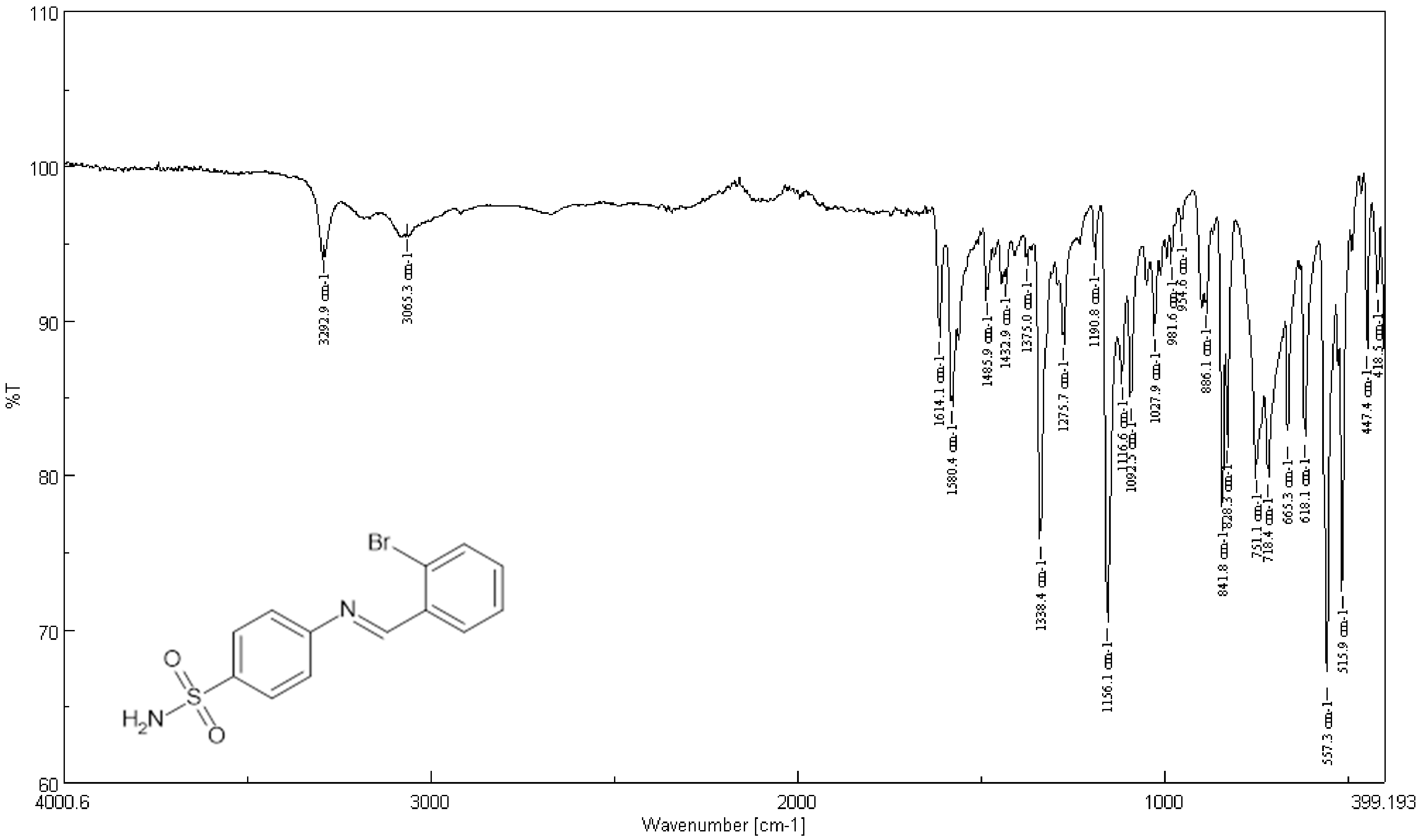

*Figure S9. FT-IR spectrum of **1b**.*

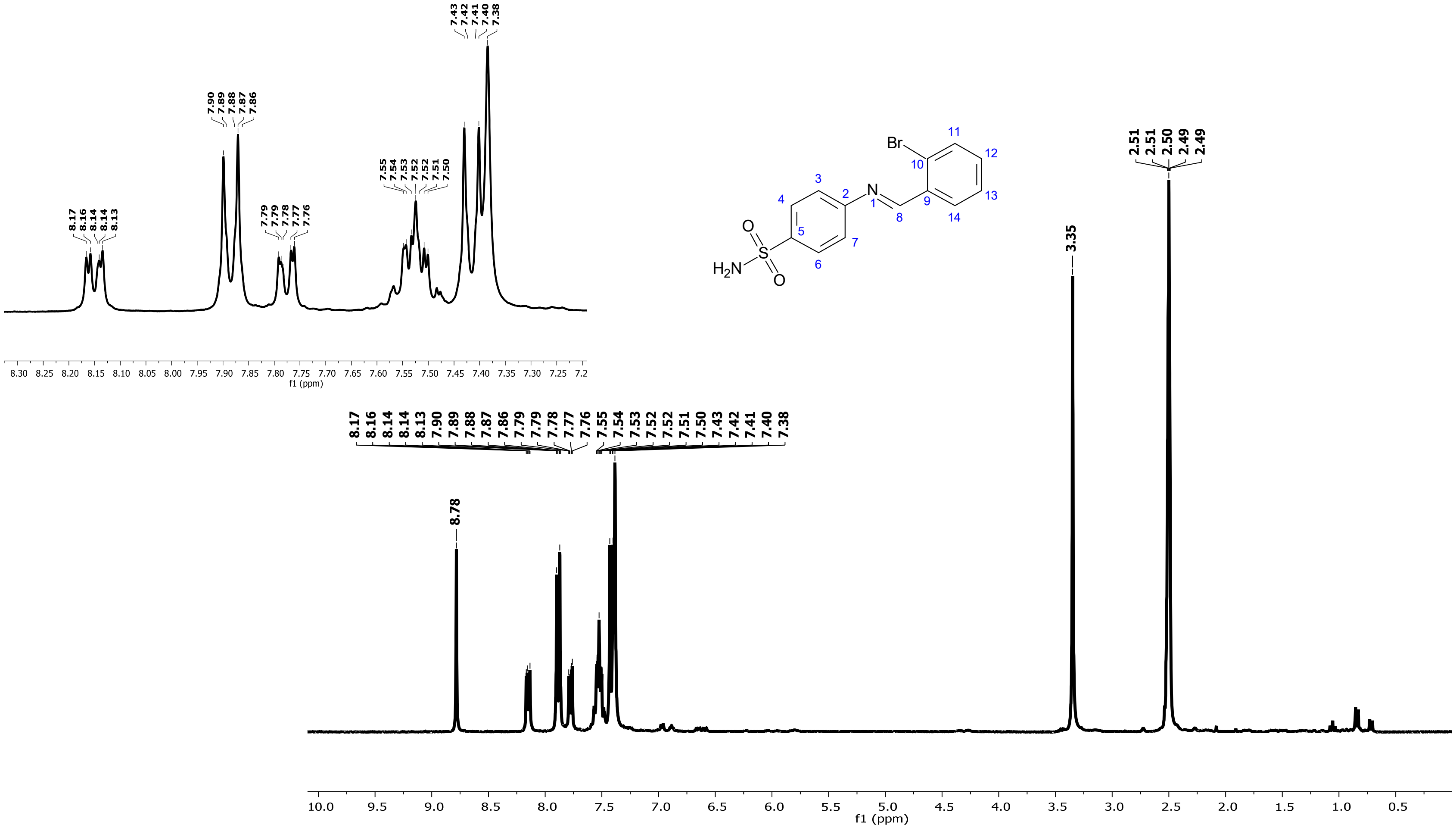

*Figure S10. ^{1}H-NMR spectrum of **1b**.*

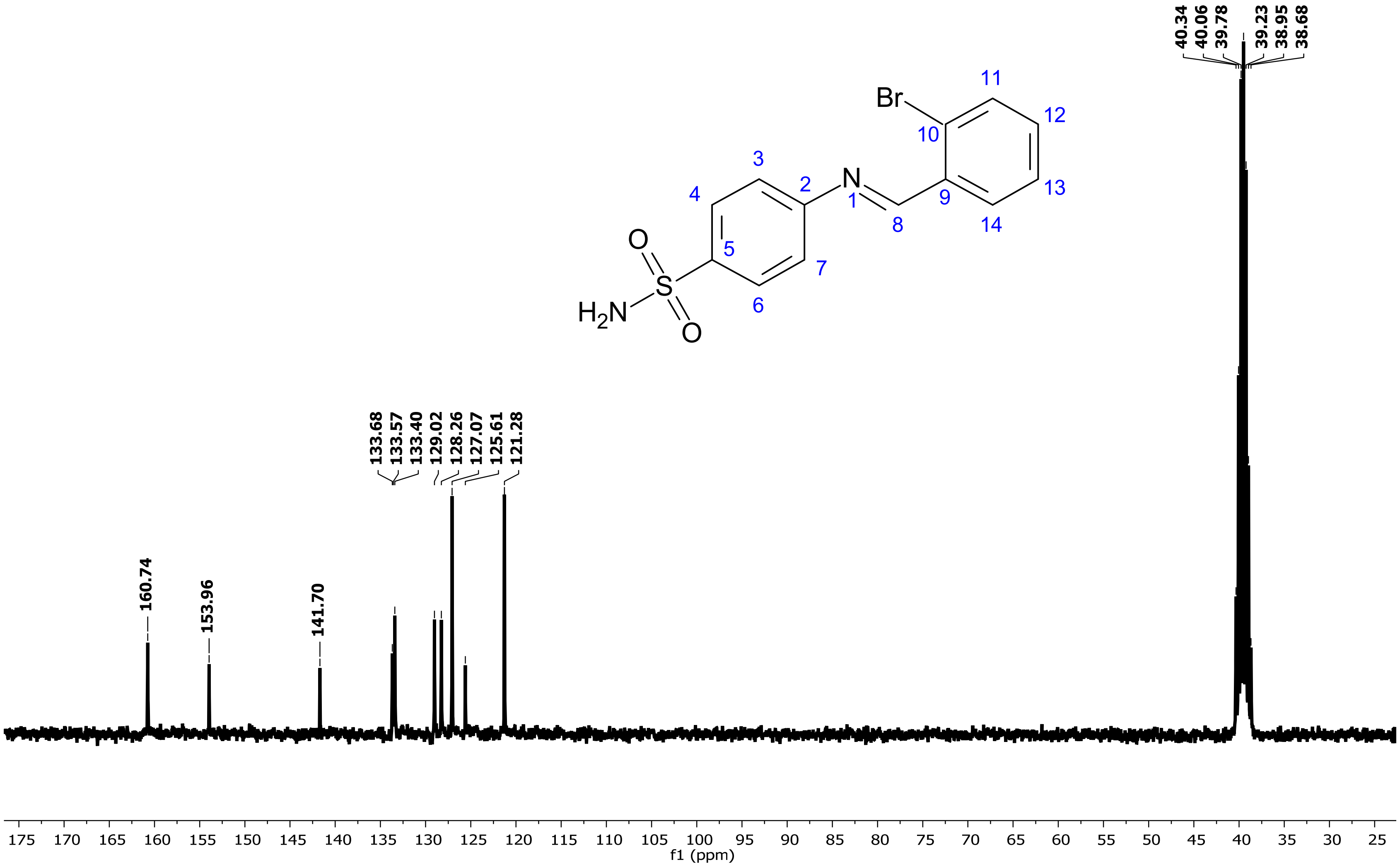

Figure S11. ^{13}*C-NMR spectrum of 1b.*

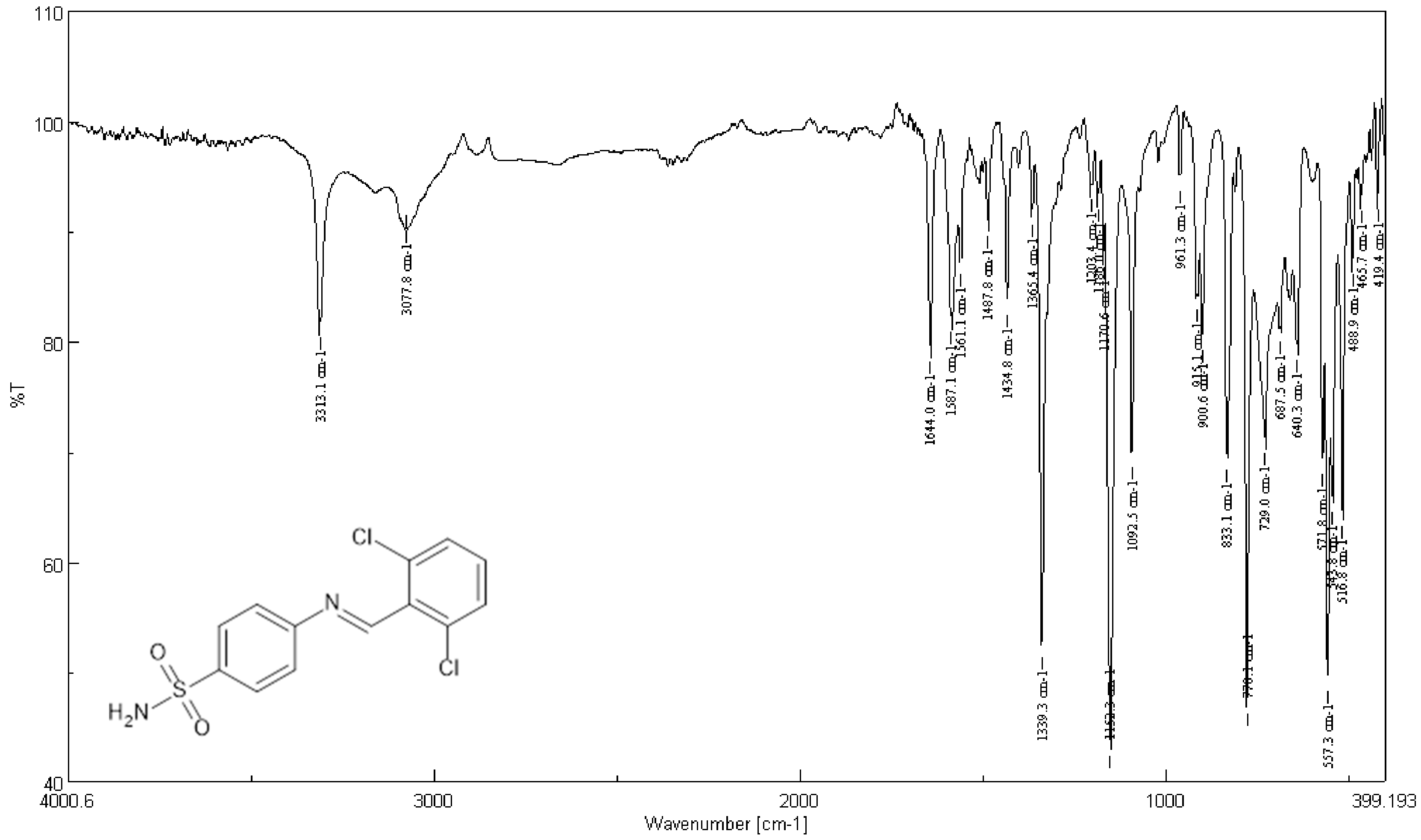

*Figure S12. FT-IR spectrum of **1c**.*

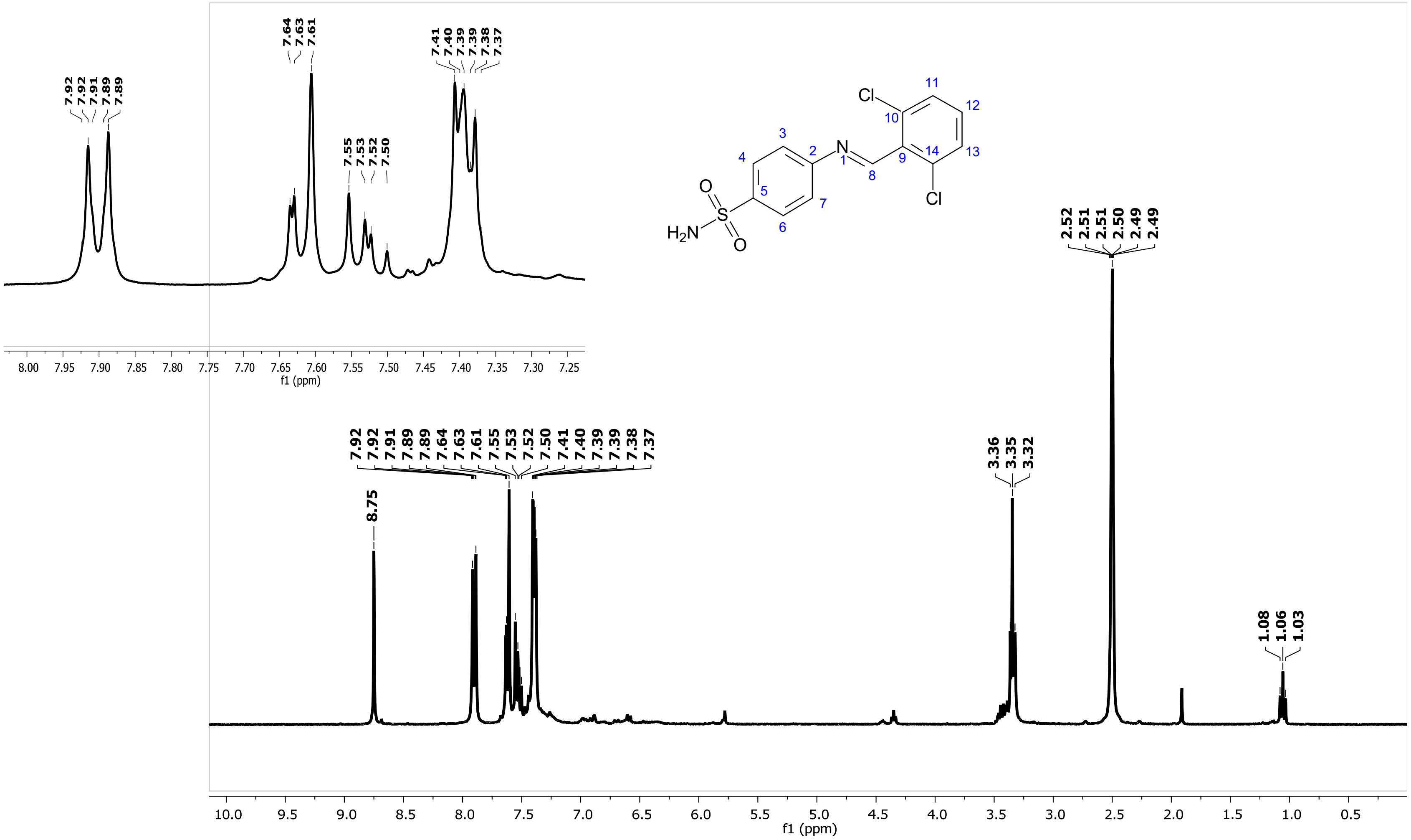

*Figure S13. ^{1}H-NMR spectrum of **1c**.*

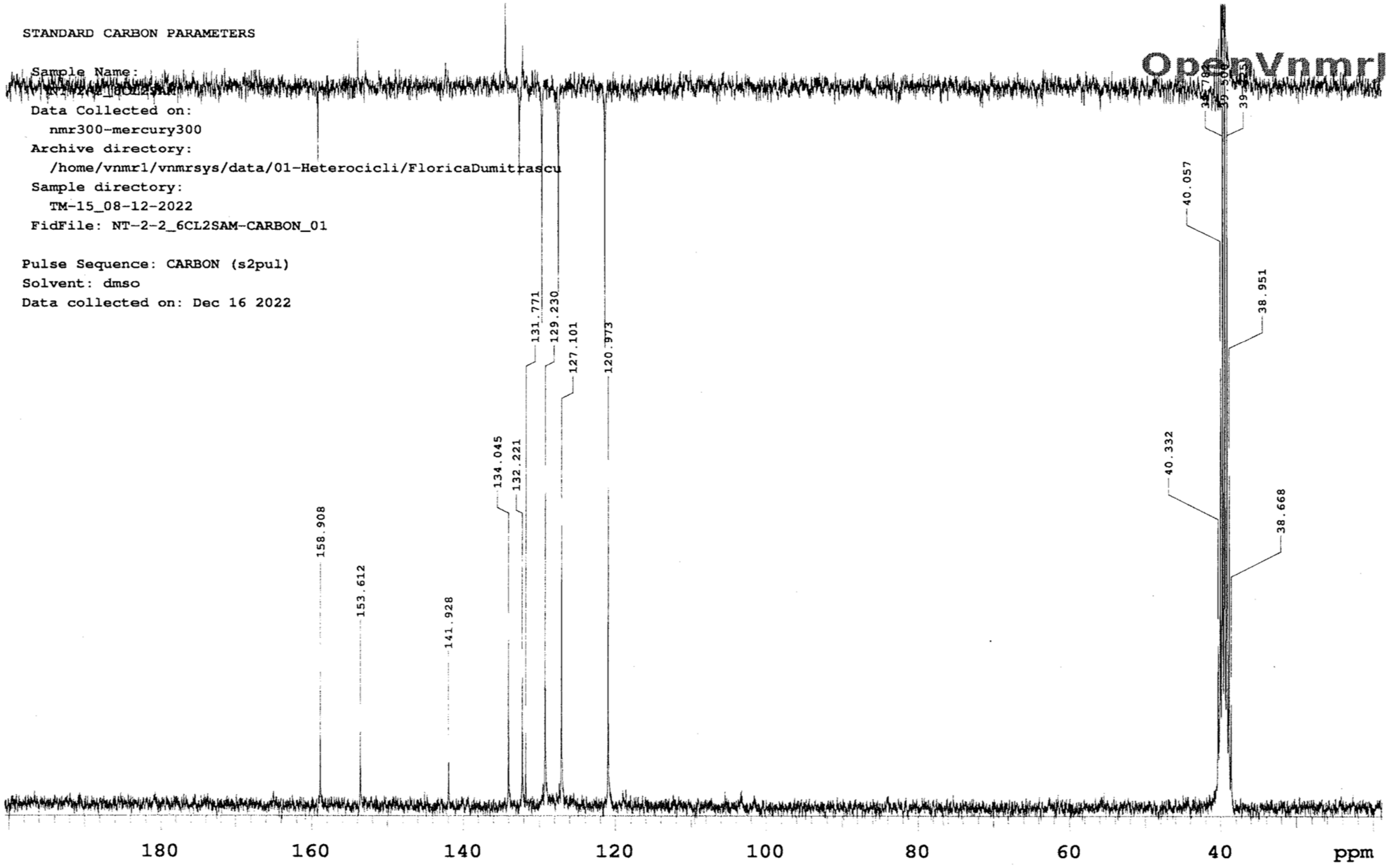

*Figure S14. ^{13}C-NMR spectrum of **1c**.*

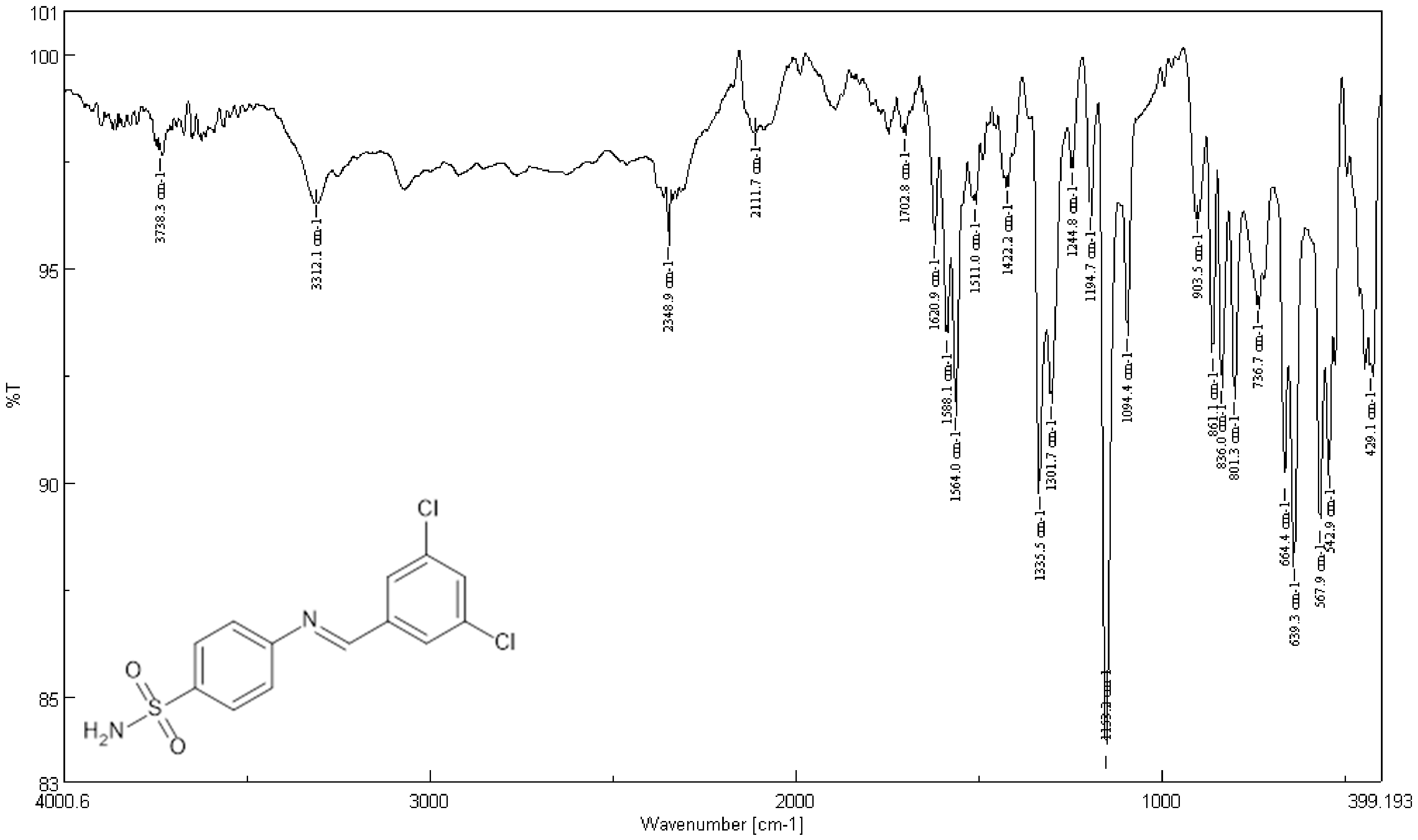

Figure S15. FT-IR spectrum of ***1d****.*

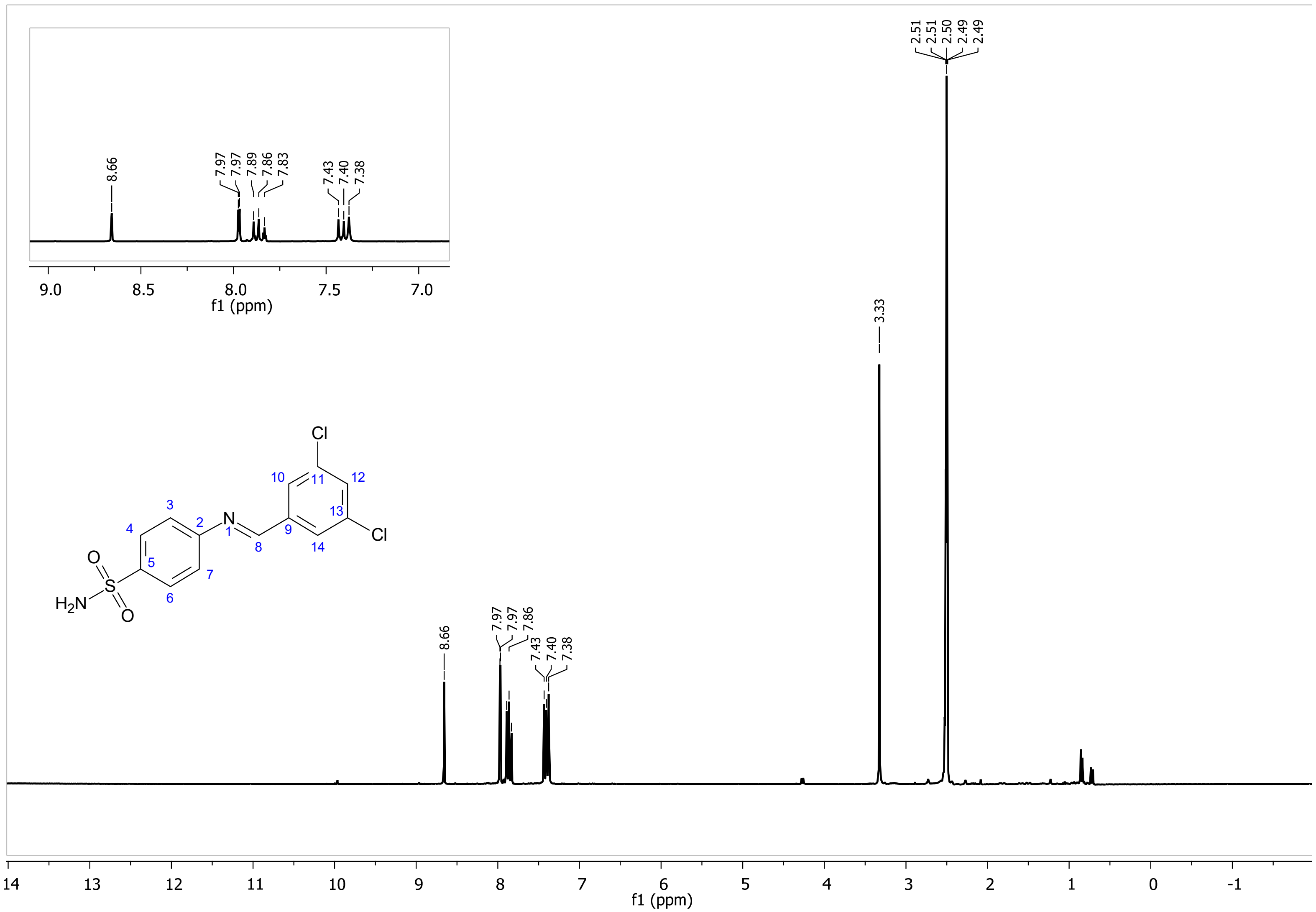

*Figure S16. ^{1}H-NMR spectrum of **1d**.*

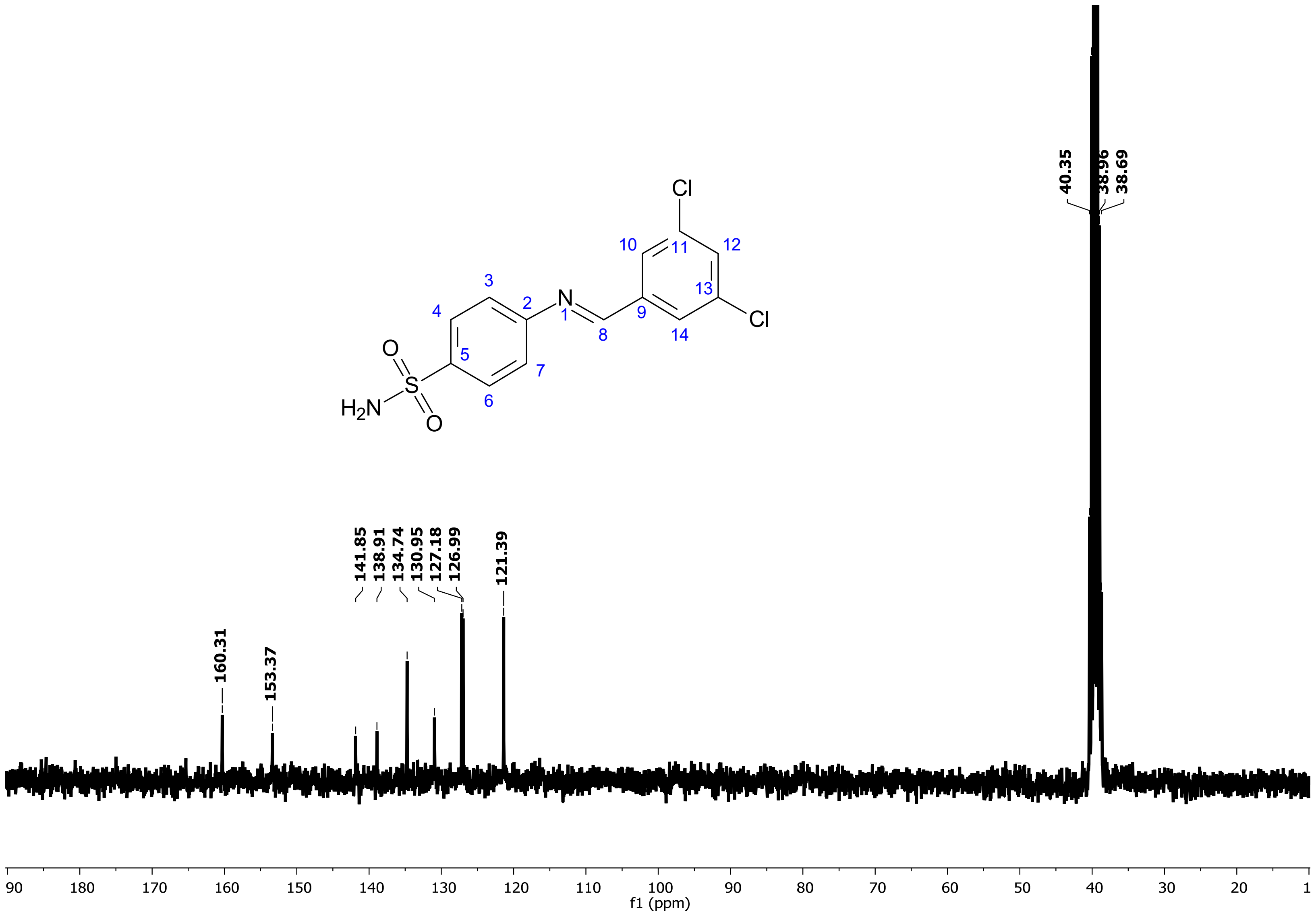

Figure S17. ^{13}C-NMR spectrum of ***1d****.*

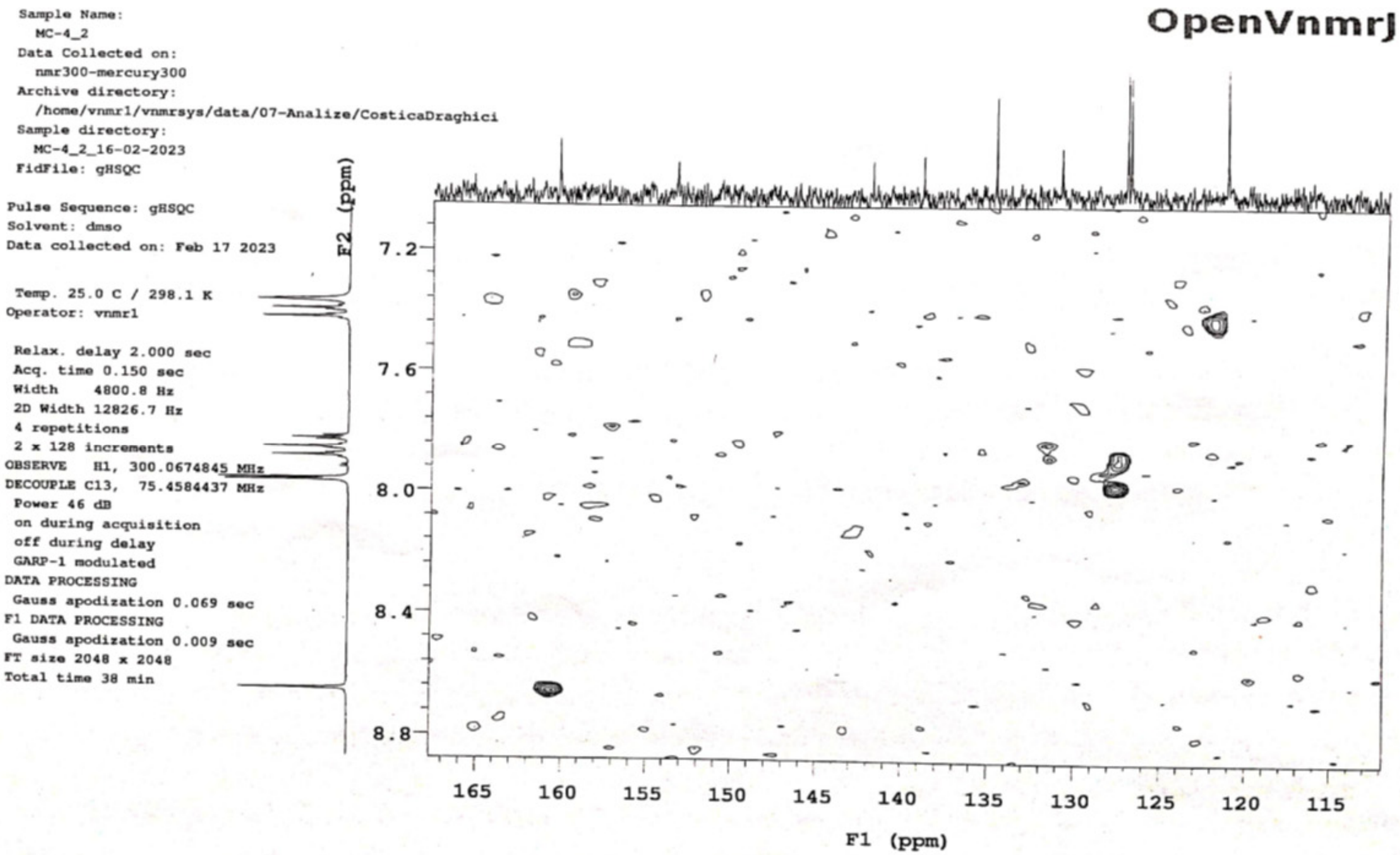

*Figure S18. HSQC spectrum of **1d**.*

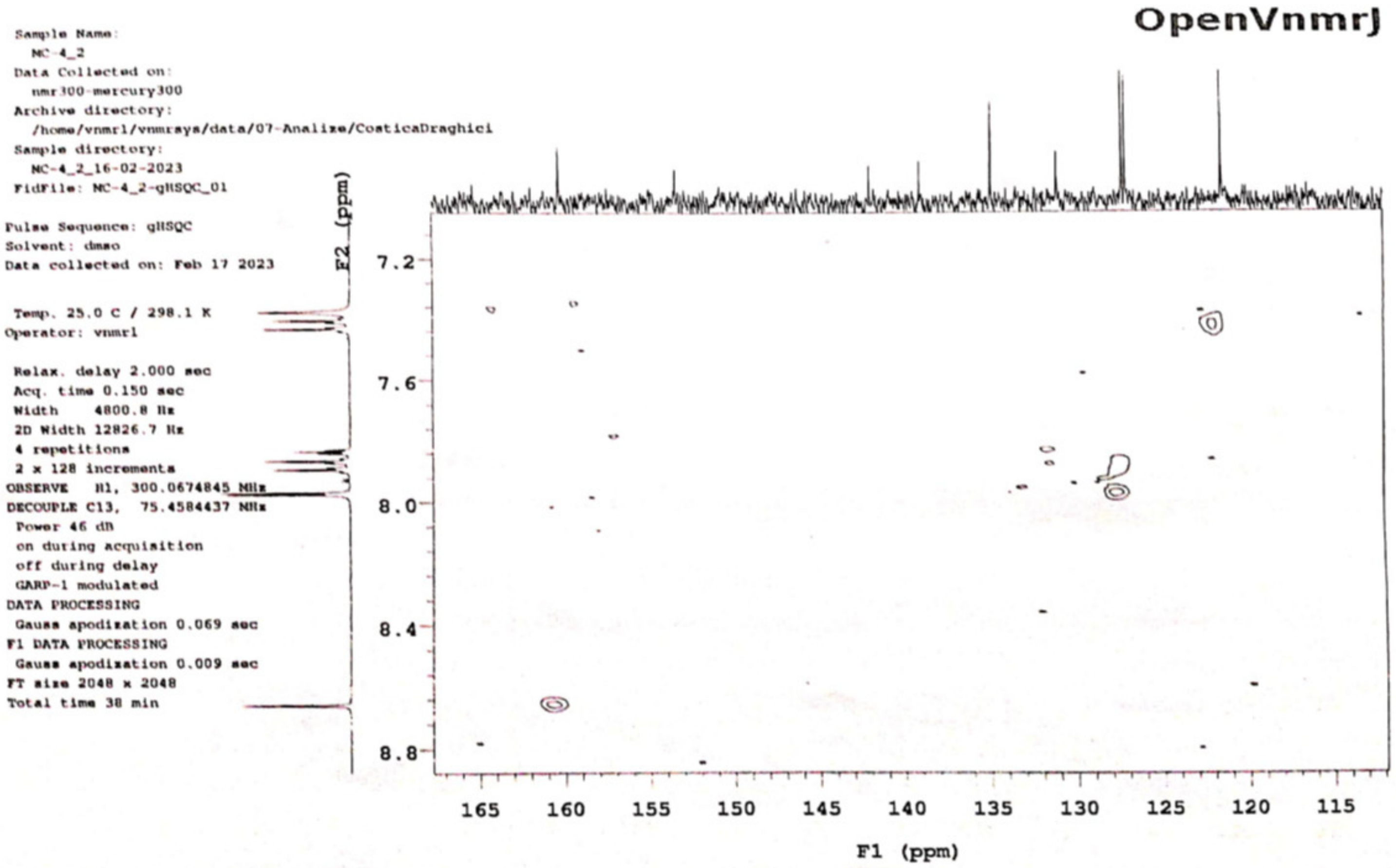

*Figure S19. HSQC spectrum of **1d**.*

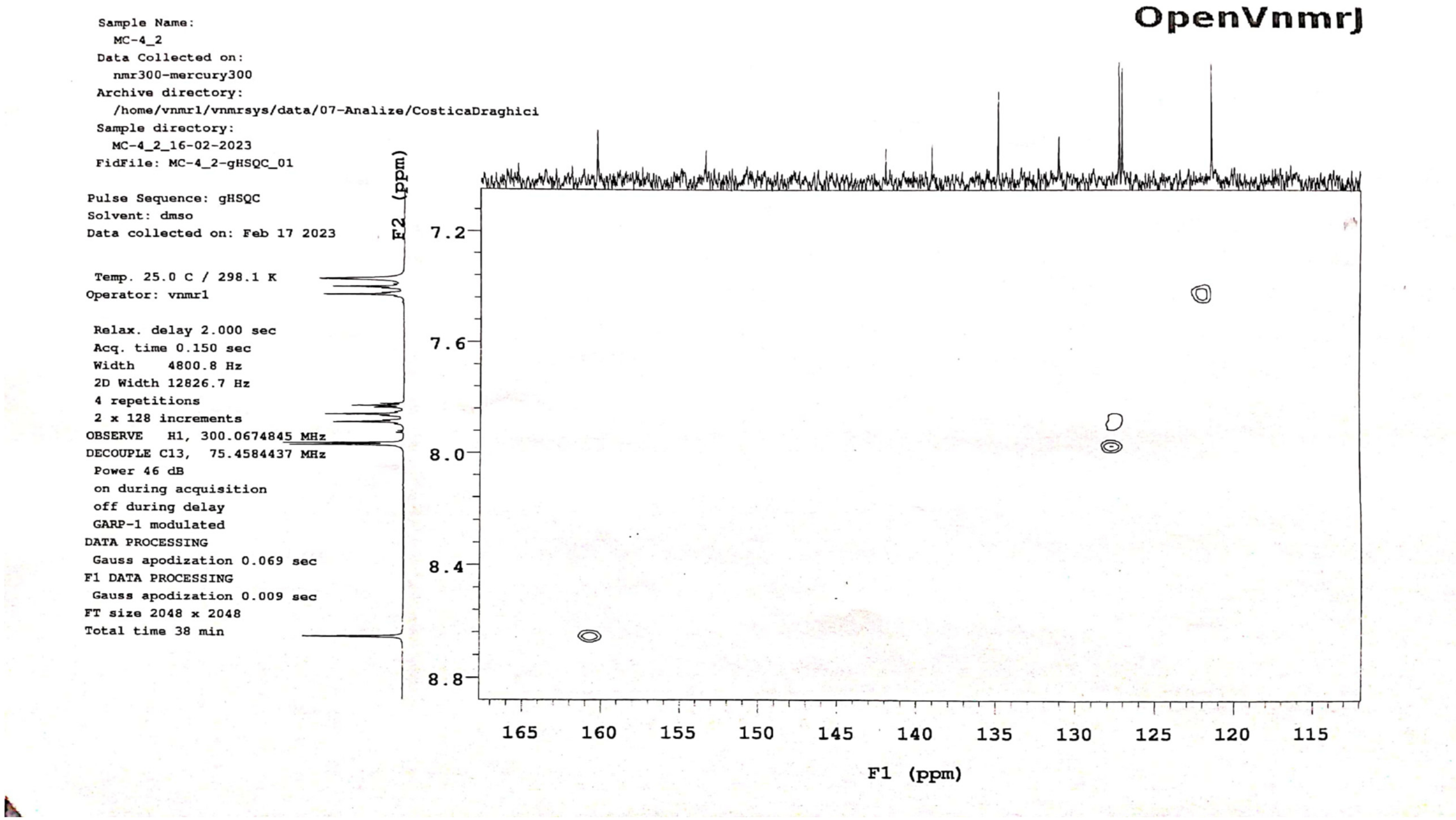

*Figure S20. HSQC spectrum of **1d**.*

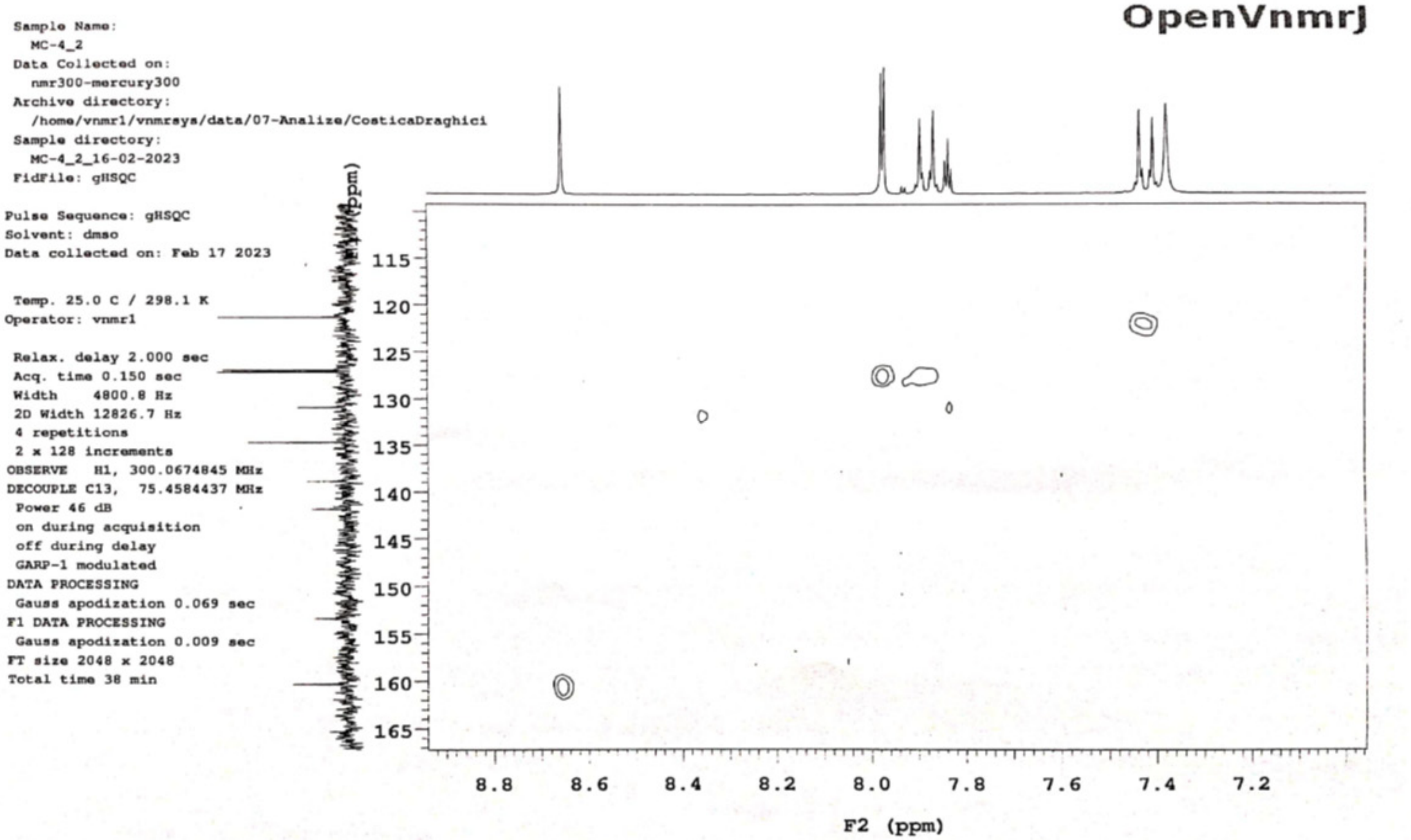

*Figure S21. HSQC spectrum of **1d**.*

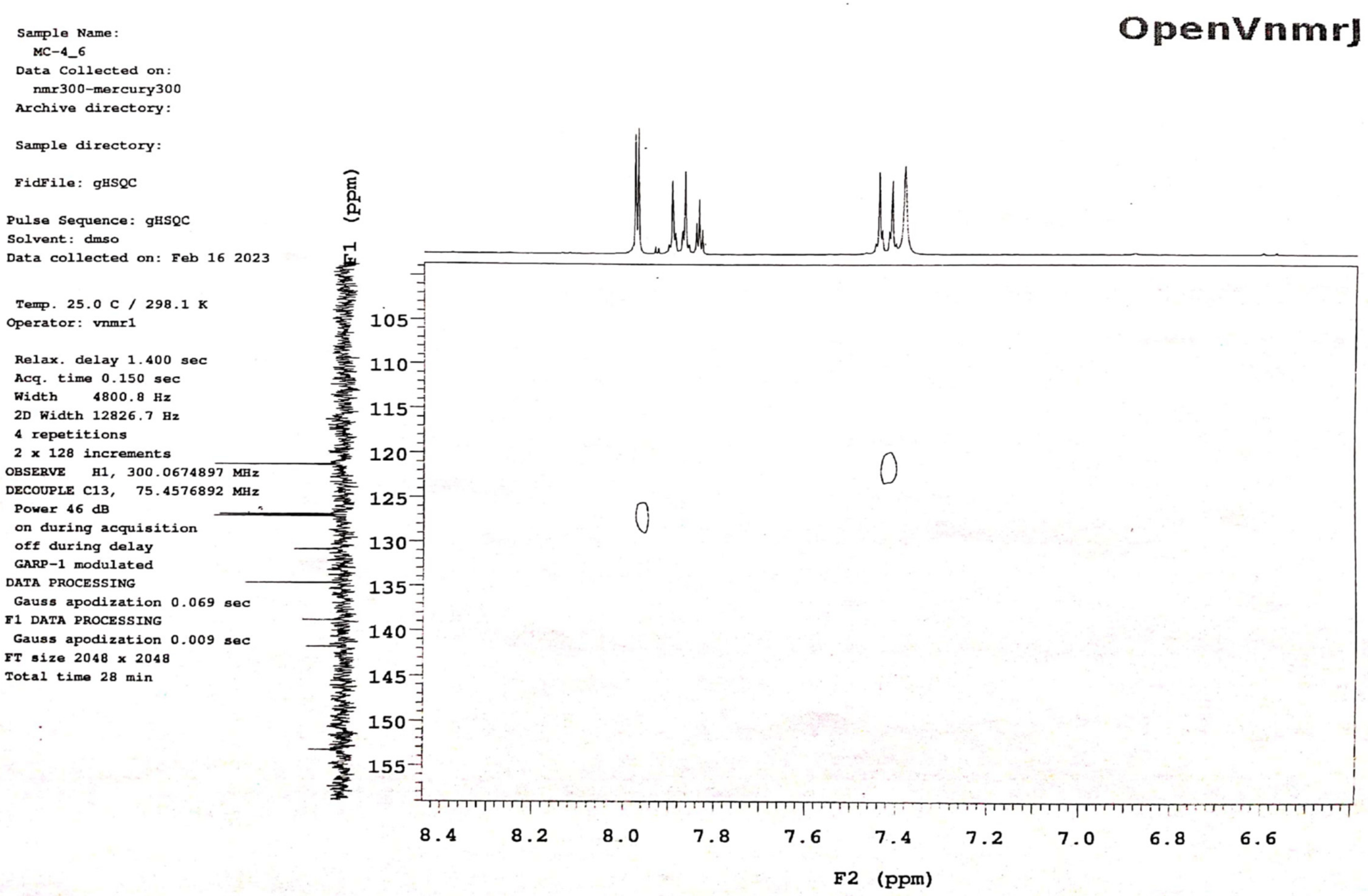

*Figure S22. HSQC spectrum of **1d**.*

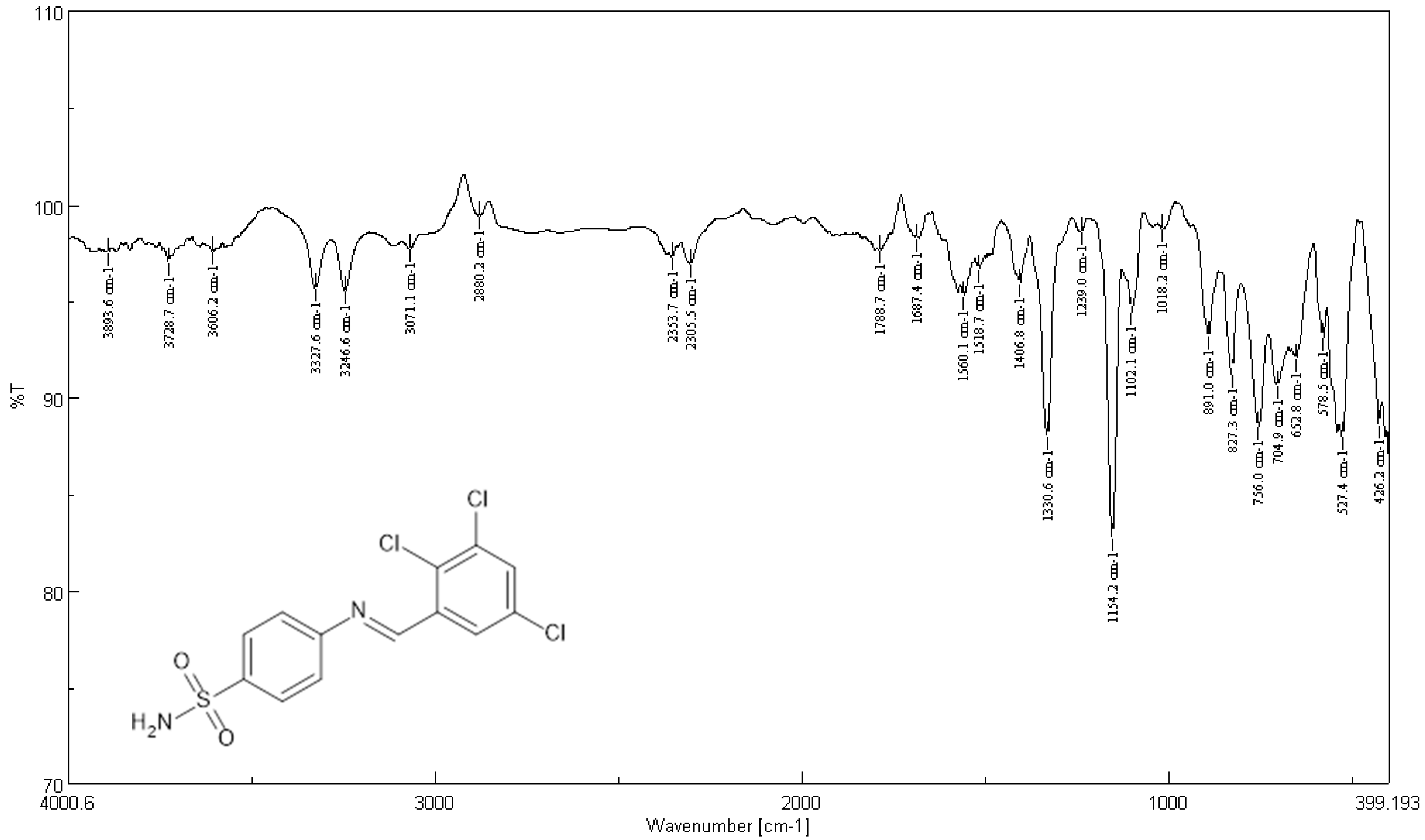

*Figure S23. FT-IR spectrum of **1e**.*

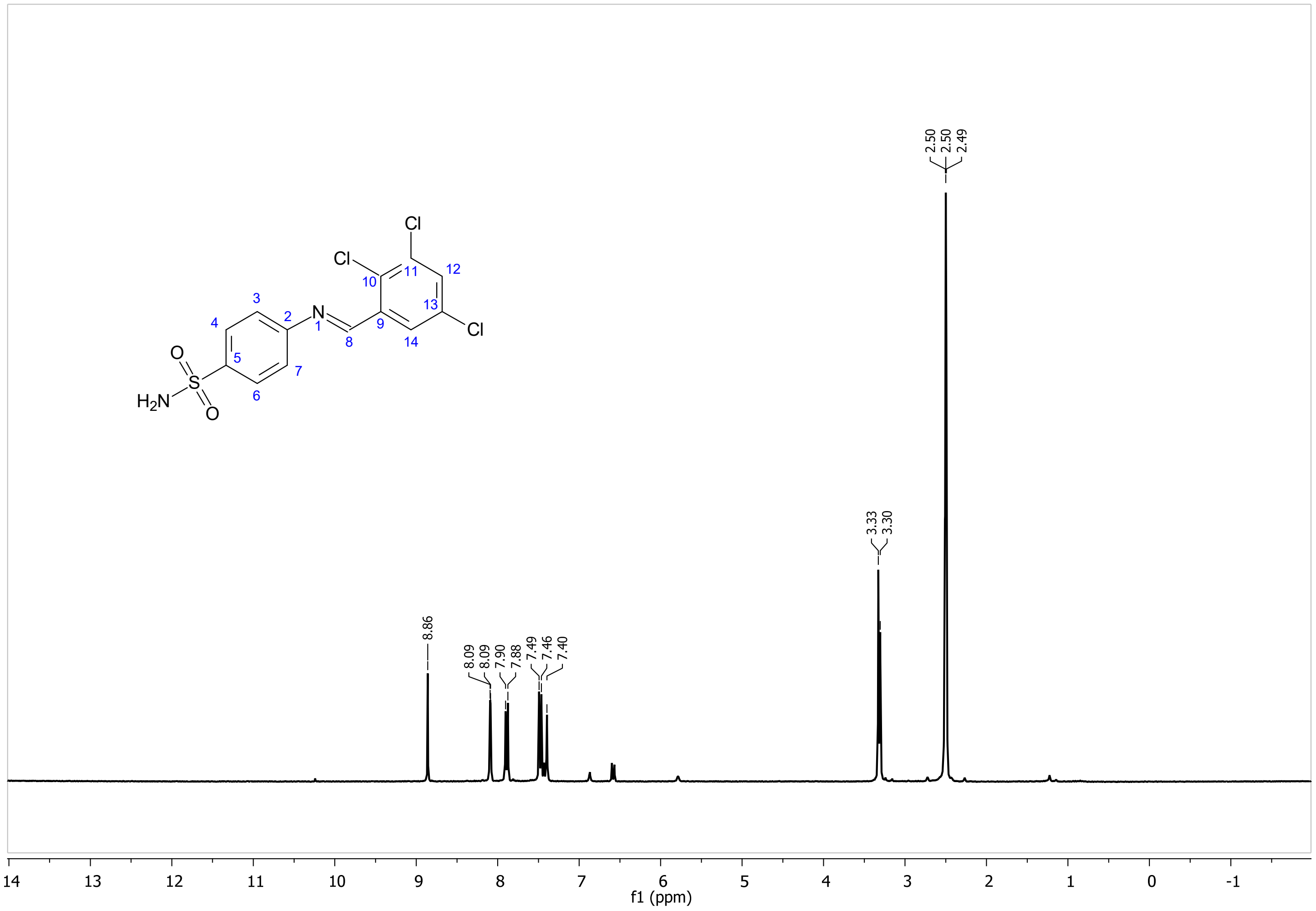

Figure S24. ^{1}*H-NMR spectrum of* ***1e***.

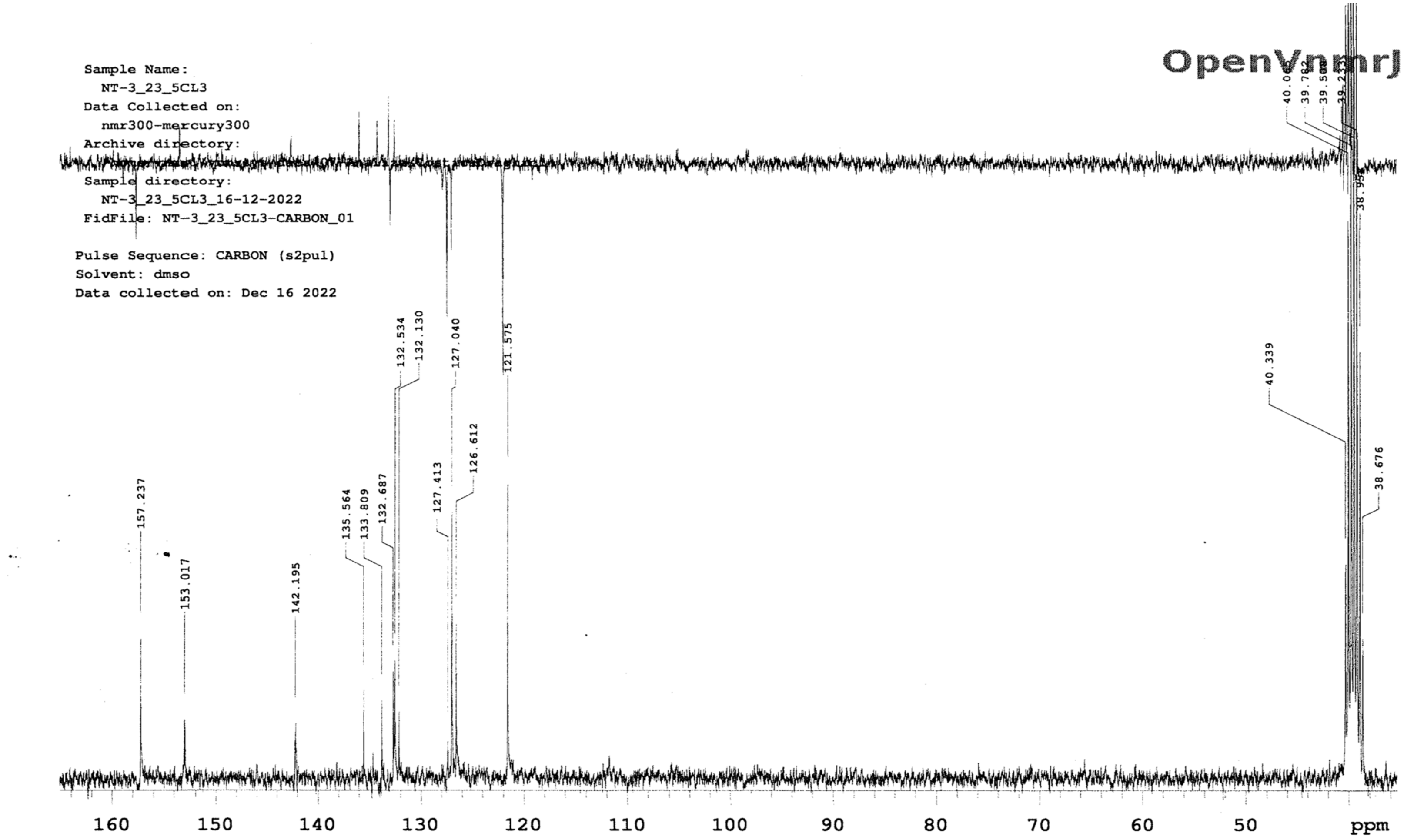

*Figure S25. ^{13}C-NMR spectrum of **1e**.*

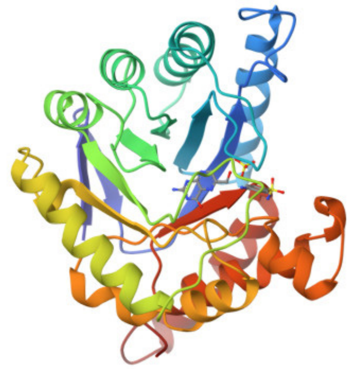

(**a**)

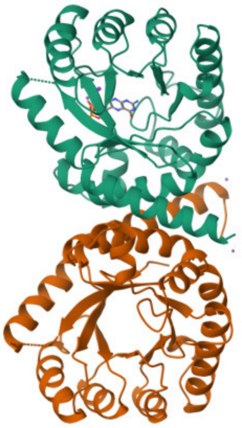

(**b**)

Figure S26. The crystal structures of ternary complex of E. coli dihydropteroate synthase (1AJ0) (a) and of S. aureus dihydropteroate synthase (1AD4) (b).

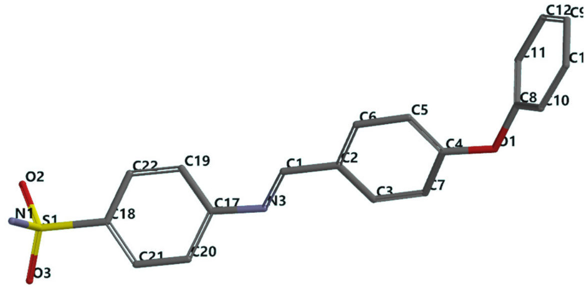

Compound 1a

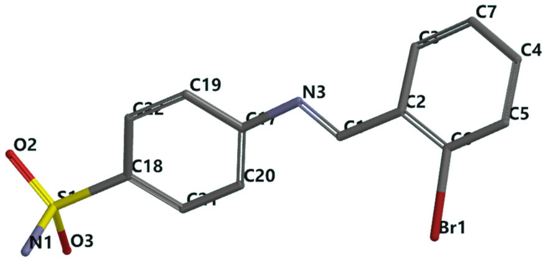

Compound 1b

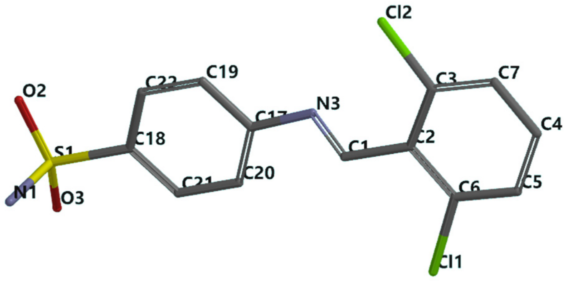

Compound 1c

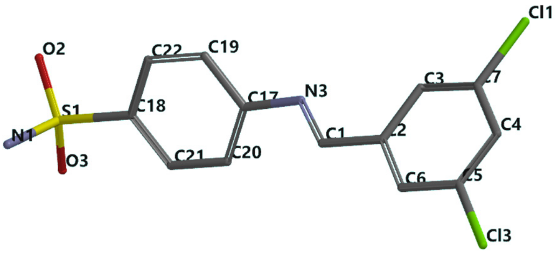

Compound 1d

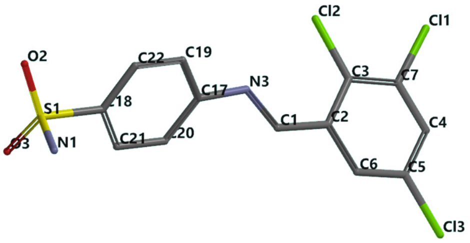

Compound 1e

Figure S27. TUBE 3D structures of compounds 1a-1e. The numbering is generated by Spartan software.

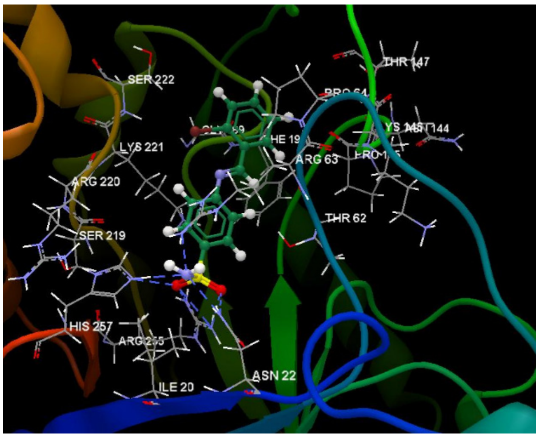

(**a**)

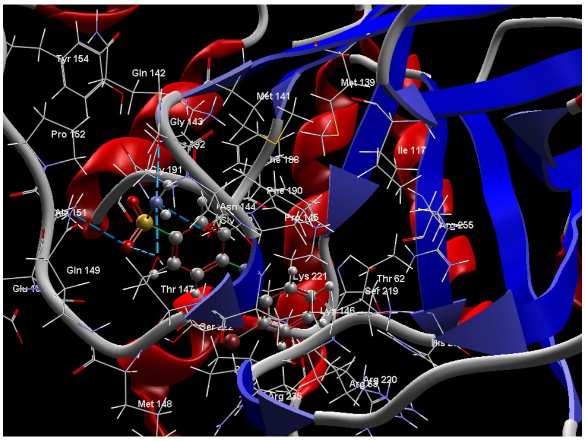

(**b**)

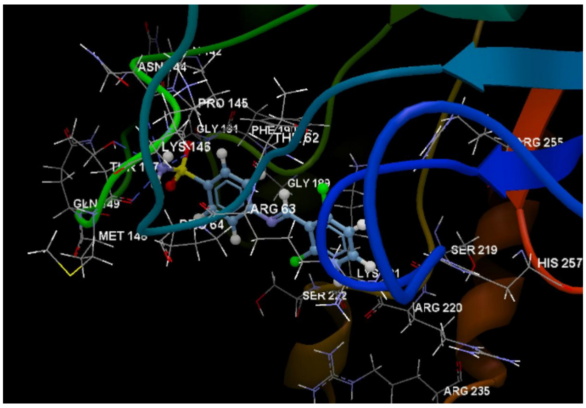

(**c**)

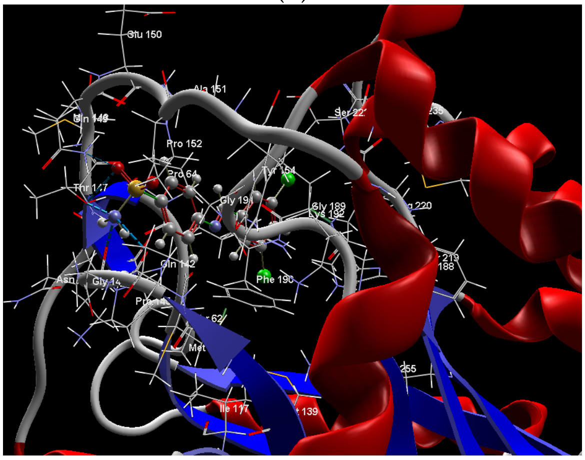

(**d**)

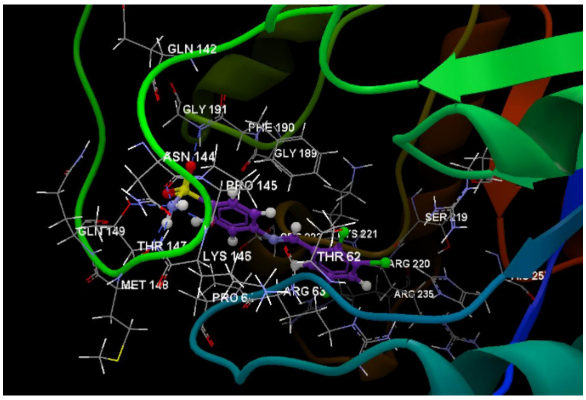

(**e**)

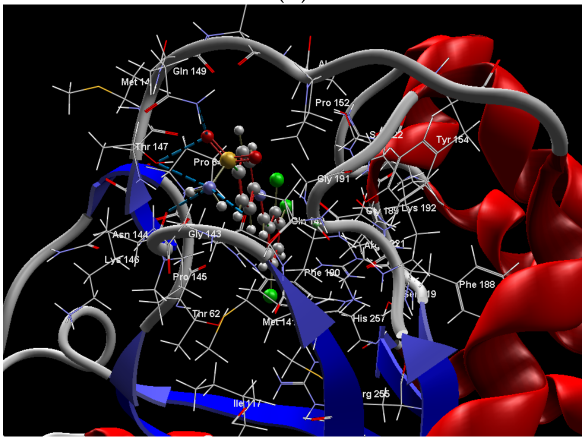

(**f**)

CLC Drug Discovery Workbench

Molegro Virtual Docker

Figure S28. Group interactions of selected compounds 1b, 1d, 1e with the active site of 1AJ0. Group interactions of 1b: (a,b); Group interactions of 1d: (c,d); Group interactions of 1e: (e,f).

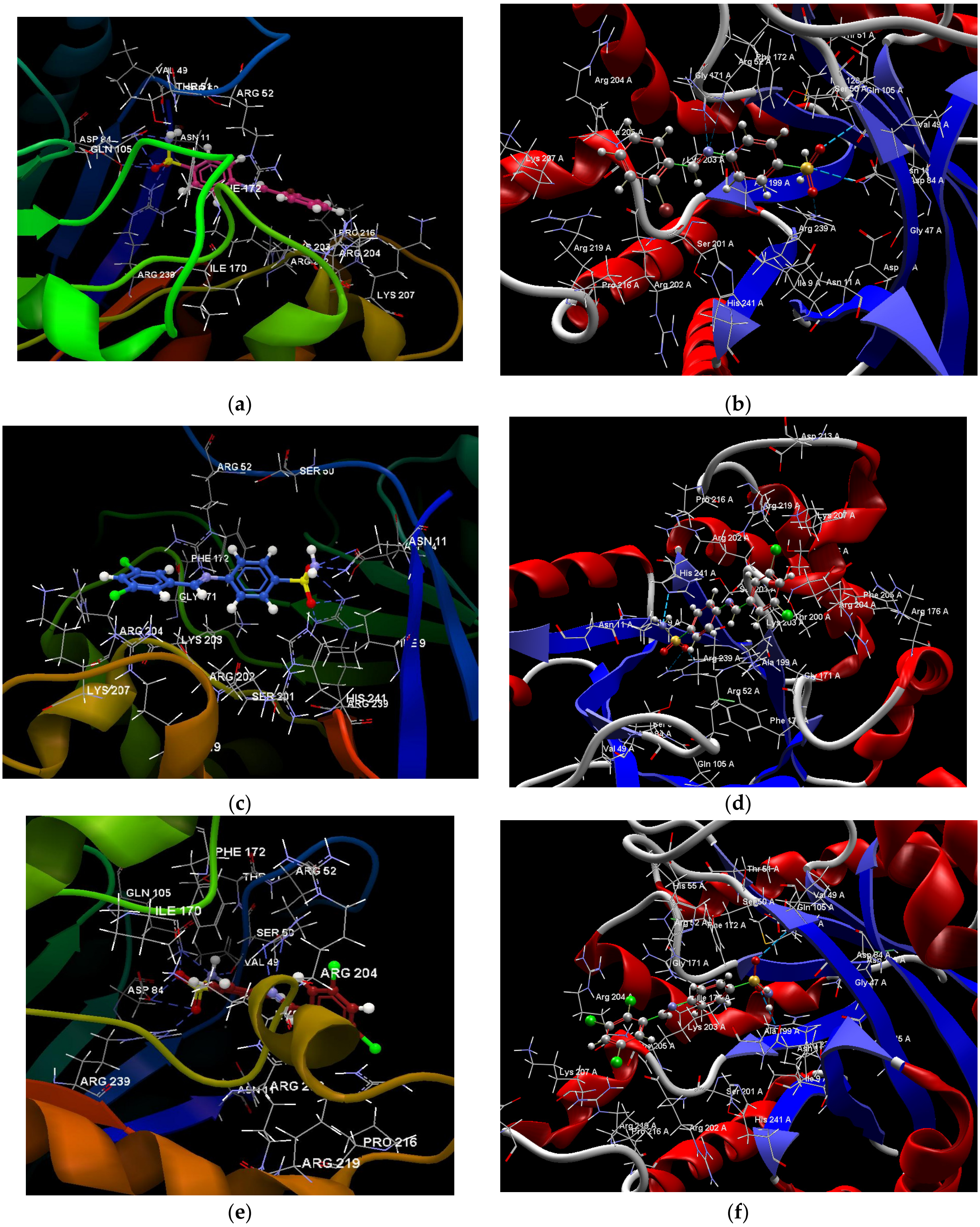

CLC Drug Discovery Workbench *Molegro Virtual Docker*

Figure S29. Group interactions of selected compounds 1b, 1d, 1e with the active site of 1AD4 (chain A). Group interactions of 1b: (a,b); Group interactions of 1d: (c,d); Group interactions of 1e: (e,f).

Table S1. Docking scores of selected ligands in the active site of 1AJ0 (CLC Drug Discovery Workbench).

Ligand	Docking Score	RMSD (Å)	Group interaction	Hydrogen bond	Bond Length (Å)
**Co-crystallized** (Sulphanilamide)	-32.36	0.07	PRO 64, ARG 663, THR 62, PRO 145, PHE 190, GLY 189, LYS 221, ARG 220, ASN 222, HIS 257, SER 219, ARG 255.	N sp^2 (N1) – O sp^2 from THR 62<br>N sp^3 (N2) – O sp^2 from SER 219<br>O sp^2 (O1) – N sp^2 from ARG 63<br>O sp^2 (O1) – N sp^2 from ARG 63<br>O sp^2 (O2) – N sp^2 from ARG 63<br>O sp^2 (O2) – N sp^2 from ARG 63	2.913<br>3.025<br>2.929<br>3.075<br>3.148<br>3.118
**Compound 1a**	-44.77	0.55	GLU 150, ALA 151, GLN 149, MET 148, THR 147, PRO 64, GLN 142, MET 141, ARG 63, GLY 143, PRO 145, LYS 146, ASN 144, THR 62, GLY 191, PHE 190, HIS 257, SER 219, GLY 189, SER 222, LYS 192, PRO 232, ARG 220, LYS 221.	N sp3 (N1) – N sp2 from GLY 191	Compound 1a
**Compound 1b**	-53.73	0.02	ASN 22, ILE 20, HIS 257, ARG 255, SER 219, ARG 220, LYS 221, SER 222, PHE 190, GLY 189, ASN 144, PRO 145, THR 62, ARG 63, THR 147, PRO 64, LYS 146.	N sp^3 (N1) – N sp^2 from ARG 63<br>N sp^3 (N1) – N sp^2 from HIS 257<br>N sp^3 (N1) – N sp^2 from ASN 22<br>O sp^2 (O2) – N sp^2 from ASN 22<br>O sp^2 (O2) – N sp^2 from ARG 255<br>O sp^2 (O3) – N sp^2 from ARG 255<br>O sp^2 (O3) – N sp^2 from ARG 255<br>O sp^2 (O3) – N sp^2 from HIS 257	2.852<br>2.920<br>2.958<br>2.883<br>3.080<br>3.294<br>2.705<br>3.167
**Compound 1c**	-53.46	0.08	GLN 142, ASN 144, PRO 145, GLY 191, LYS 146, THR 147, GLN 149, MET 148, THR 62, PHE 190, GLY 189, ARG 63, PRO 64, ARG 255, SER 219, HIS 257, ARG 220, ARG 235, SER 222, LYS 221.	N sp^3 (N1) – O sp^2 from THR 147<br>N sp^3 (N1) – N sp^2 from THR 147<br>N sp^3 (N1) – O sp^3 from THR 147<br>N sp^3 (N1) – O sp^2 from ASN144<br>N sp^3 (N1) – O sp^2 from PRO 145<br>O sp^2 (O2) – N sp^2 from GLY 191	2.570<br>3.212<br>3.012<br>3.103<br>2.667<br>3.067
**Compound 1d**	-53.60	0.10	ASN 144, THR 147, GLY 143, GLN 149, GLN 142, MET 141, GLY 191, PHE 190, GLY 189, PRO 145, PRO 64, THR 62, ARG 63, ILE 20, ARG 255, HIS 257, SER 219, ARG 220, LYS 221, MET 218	N sp^3 (N1) – O sp^2 from SER 219<br>O sp^2 (O2) – N sp^2 from ARG 255<br>O sp^2 (O3) – N sp^2 from ARG 63<br>O sp^2 (O3) – N sp^2 from ARG 63	3.073<br>3.072<br>3.177<br>2.877

**Compound 1e**	-51.75	0.04	GLN 142, GLY 191, PHE 190, ASN 144, GLY 189, PRO 145, GLN 149, THR 147, MET 148, LYS 146, PRO 64, ARG 63, ARG 235, HIS 257, ARG 220, SER 219, ARG 255, LYS 221, SER 222, THR 62.	N sp^3 (N1) – O sp^2 from THR 147<br>N sp^3 (N1) – N sp^2 from THR 147<br>N sp^3 (N1) – O sp^3 from THR 147<br>N sp^3 (N1) – O sp^2 from ASN144<br>N sp^3 (N1) – O sp^2 from PRO 145<br>O sp^2 (O2) – N sp^2 from GLY 191	2.757<br>3.214<br>2.753<br>2.810<br>2.769<br>2.926

Table S2. Docking scores of selected ligands in the active site of 1AJ0 (Molegro Virtual Docker).

Ligand	Mol Dock Score	Molecule Contributions	Hydrogen bond	Bond Length Å	Steric Interactions	Distance Å
**Co-crystallized (Sulphanilamide)**	-61.30	ARG 63, ASN 144, GLN 142, GLN149, GLY 189, GLY 191, LYS 146, MET 141, PHE 180, PRO 64, PRO 145, THR 62, THR 147.	N sp^2 (N2) - O sp^2 from THR 147<br>N sp^2 (N2) - O sp^3 from THR 147<br>N sp^2 (N2) - O sp^2 from ASN 144<br>N sp^2 (N2) - O sp^2 from PRO145	2.880<br>2.897<br>2.926<br>2.873	C sp^2 (C3)- O sp^2 from PRO145<br>O sp^2 (O1)- N sp^2 from GLY 191<br>O sp^2 (O2)- N sp^2 from GLY 191	2.844<br>3.166<br>3.084
**Compound 1a**	-128.56	ALA 151, ARG 63, ARG 255, ASN 22, ASN 144, GLN 142, GLN 149, GLU 150, GLY 143, GLY 189, GLY 191, HIS 257, ILE 20, LYS 146, LYS 221, MET 148, PHE 190, PRO 64, PRO 145, PRO 152, SER 61, SER 219, THR 62, THR 147.	N sp^2 (N1) - O sp^2 from THR 147<br>N sp^2 (N1) - O sp^3 from THR 147<br>N sp^2 (N1) - O sp^2 from GLN 149<br>O sp^2 (O2) - O sp^3 from THR 147<br>O sp^2 (O3) - N sp^2 from ARG 63	3.070<br>2.726<br>2.865<br>3.012<br>3.506	C sp^2 (C19)- O sp^2 from PRO145<br>C sp^2 (C19)- C sp^2 from PHE 190	3.144<br>3.123
**Compound 1b**	-105.55	ALA 151, ARG 63, ASN 144, GLN 142, GLN 149, GLU 150, GLY 143, GLY 189, GLY 191, LYS 146, LYS 221, MET 148, PHE 190, PRO 64, PRO 145, PRO 152, SER 222, THR 62, THR 147.	N sp^2 (N1) - O sp^2 from ASN 144<br>N sp^2 (N1) - O sp^3 from THR 147<br>N sp^2 (N1) - O sp^2 from GLN 142<br>O sp^2 (O2) - O sp^3 from THR 147	2.778<br>2.903<br>2.963<br>2.753<br>3.132	C sp^2 (C17)- O sp^2 from PRO145<br>C sp^2 (C19)- O sp^2 from PRO145<br>C sp^2 (C19)- C sp^2 from PHE 190<br>N sp^2 (N1) - N sp^2 from ASN 144	3.049<br>3.083<br>3.024<br>3.128<br>2.633

			O sp^2 (O3) - N sp^2 from GLN 149		O sp^3 (O2) - C sp^3 from GLY 191	
**Compound 1c**	-96.22	ARG 220, ARG 235, ASN 144, GLN 142, GLN 149, GLY 189, GLY 191 HIS257, LYS 146, LYS 221, MET 148, PHE 190, PRO 64, PRO 145, SER 219, SER 222, THR 62, THR 147.	N sp^2 (N1) - O sp^2 from THR 147<br>N sp^2 (N1) - O sp^3 from THR 147<br>N sp^2 (N1) - O sp^2 from ASN 144<br>N sp^2 (N1) - O sp^2 from PRO 145<br>O sp^2 (O3) - N sp^2 from GLY 191	2.597<br>2.952<br>3.038<br>2.678<br>2.912	C sp^2 (C21)- O sp^2 from PRO145	2.720
**Compound 1d**	-16.29	ALA 151, ARG 63, ARG 220, ASN 144, GLN 142, GLN 149, GLU 150, GLY 143, GLY 189, GLY 191, LYS 146, LYS 221, MET 141, PHE 190, PRO 64, PRO 145, PRO 152, SER 222, THR 62, THR 147.	N sp^2 (N1) - O sp^3 from THR 147<br>N sp^2 (N1) - O sp^2 from GLN 142<br>N sp^2 (N1) - O sp^2 from ASN 144<br>O sp^2 (O2) - O sp^3 from THR 147<br>O sp^2 (O3) - N sp^2 from GLN 149	2.885<br>3.017<br>3.011<br>2.686<br>2.887	N sp^2 (N1) - N sp^2 from ASN 144<br>O sp^2 (O2) - C sp^2 from GLN 149<br>O sp^2 (O3) - C sp^3 from GLY 191<br>C sp^2 (C17)- O sp^2 from PRO145	3.146<br>3.171<br>2.948<br>3.131
**Compound 1e**	-14.94	ALA 151, ARG 63, ASN 144, GLN 142, GLN 149, GLU 150, GLY 143, GLY 189, GLY 191, LYS 146, LYS 221, PHE 190, PRO 64, PRO 145, PRO 152, SER 222, THR 62, THR 147.	N sp^2 (N1) - O sp^3 from THR 147<br>N sp^2 (N1) - O sp^2 from GLN 142<br>N sp^2 (N1) - O sp^2 from ASN 144<br>O sp^2 (O2) - O sp^3 from THR 147<br>O sp^2 (O2) - N sp^2 from GLN 149<br>O sp^2 (O3) - N sp^2 from GLY 191	3.002<br>2.972<br>2.682<br>2.705<br>3.054<br>3.087	O sp^2 (O3) - C sp^3 from GLY 191<br>C sp^2 (C17)- O sp^2 from PRO145	2.611<br>3.110

Table S3. Docking scores of selected ligands in the active site of 1AD4 (CLC Drug Discovery Workbench).

Ligand	Docking Score	RMSD (Å)	Group interaction	Hydrogen bond	Bond Length (Å)
**Co-crystallized** ($OH-CH_2$-pterin-pyrophosphate)	-41.71	1.40	VAL 162, ASN 103, ASP 104, ASP 84, VAL 49, ASN 11, ILE 9, SER 50, GLN 105, HIS 241, ARG 239, MET 128, LEU 197, ASP 167, ALA 199, GLY 169, ILE 170, PHE 172, LYS 203, ARG 52.	N sp^2 (N6) – O sp^2 from ASP 103<br>N sp^2 (N6) – O sp^2 from ASP 167<br>N sp^2 (N5) – N sp^2 from ASP 103<br>N sp^2 (N7) – O sp^2 from ASP 167<br>N sp^2 (N4) – O sp^3 from ASP 64<br>N sp^2 (N4) – N sp^2 from GLN 105<br>N sp^2 (N4) – N sp^3 from LYS 203<br>O sp^2 (O5P) – N sp^2 from ASN 11<br>O sp^2 (O5P) – N sp^2 from HIS 241<br>O sp^2 (O5P) – N sp^2 from ARG 239<br>O sp^2 (O6P) – N sp^2 from ARG 239	2.445<br>3.046<br>3.159<br>2.639<br>2.738<br>3.165<br>3.096<br>2.878<br>2.904<br>2.920<br>2.844
**Compound 1a**	-43.56	0.36	ASN 11, ILE 9, HIS 241, PRO 216, SER 201, ARG 202, LYS 203, ARG 204, LYS 207, ARG 219, ASP 84, ASN 103, ASP 104, GLN 105, MET 128, ASP 164, ARG 239, ARG 52, ALA 199, PHE 172, GLY 171, ILE 170.	N sp^3 (N1) – N sp^2 from ARG 219<br>O sp^2 (O2) – N sp^2 from ARG 219<br>O sp^2 (O2) – N sp^2 from ARG 219<br>N sp^2 (N3) – N sp^2 from ARG 52<br>O sp^3 (O1) – N sp^2 from ARG 239<br>O sp^3 (O1) – N sp^2 from ARG 239	3.073<br>2.695<br>3.392<br>2.774<br>3.152<br>3.046
**Compound 1b**	-41.41	0.09	ARG 52, THR 51, SER 50, VAL 49, PHE 172, GLN 105, ASP 94, ARG 239, ILE 170, ASN 11, ARG 204, LYS 203, ARG 202, ARG 219, LYS 207, PRO 216.	N sp^3 (N1) – O sp^2 from VAL 49<br>N sp^3 (N1) – N sp^2 from GLN 105<br>O sp^2 (O2) – N sp^2 from GLN 105<br>O sp^2 (O2) – O sp^3 from ASP 84<br>O sp^2 (O3) - O sp^3 from ASP 84<br>O sp^2 (O3) – N sp^2 from ARG 239<br>N sp^2 (N3) – N sp^2 from ARG 52	2.809<br>2.796<br>3.054<br>3.065<br>3.137<br>2.576<br>2.730
**Compound 1c**	-34.13	0.24	ARG 52, THR 51, SER 50, VAL 49, ASN 11, ASP 84, GLN 105, PHE 172, PRO 216, ARG 204, LYS 203, ARG 202, ARG 219, SER 201, ARG 239, HIS 241, ILE 9, ASN 103.	N sp^3 (N1) – O sp^2 from GLN 105<br>N sp^3 (N1) – N sp^2 from GLN 105<br>O sp^2 (O2) – N sp^2 from ARG 239<br>O sp^2 (O2) – O sp^3 from ASP 84<br>O sp^2 (O3) - O sp^3 from ASP 84<br>O sp^2 (O3) – N sp^2 from ARG 239	3.043<br>2.651<br>2.957<br>2.878<br>3.314<br>3.199

				O sp^2 (O3) – N sp^2 from ARG 239	3.035
**Compound 1d**	-41.83	0.21	ARG 52, SER 50, ASN 11, ASP 84, PHE 172, GLY 171, ARG 204, LYS 203, LYS 207, ARG 202, ARG 219, SER 201, ARG 239, HIS 241, ILE 9.	N sp^3 (N1) – O sp^2 from ASN 11<br>N sp^3 (N1) – N sp^2 from ASN 11<br>O sp^2 (O2) – N sp^2 from ARG 239<br>O sp^2 (O2) – N sp^2 from ARG 239<br>O sp^2 (O3) – N sp^2 from ARG 239<br>N sp^2 (N3) – N sp^2 from ARG 52	2.694<br>2.980<br>2.951<br>3.146<br>3.045<br>2.900
**Compound 1e**	-38.17	0.03	ARG 52, THR 51, SER 50, VAL 49, PHE 172, GLN 105, ASP 94, ARG 239, ILE 170, ASN 11, ARG 204, LYS 203, ARG 202, ARG 219, PRO 216.	N sp^3 (N1) – O sp^2 from VAL 49<br>N sp^3 (N1) – N sp^2 from GLN 105<br>O sp^2 (O2) – N sp^2 from GLN 105<br>O sp^2 (O2) – O sp^3 from ASP 84<br>O sp^2 (O3) - O sp^3 from ASP 84<br>O sp^2 (O3) – N sp^2 from ARG 239<br>N sp^2 (N3) – N sp^2 from ARG 52<br>N sp^2 (N3) – N sp^2 from ARG 52	2.730<br>2.564<br>2.976<br>3.148<br>2.952<br>2.628<br>2.944<br>3.322

Table S4. Docking scores of selected ligands in the active site of 1AJ0 (Molegro Virtual Docker).

**Ligand**	**Mol Dock Score**	**Molecule Contributions**	**Hydrogen bond**	**Bond Length (Å)**	**Electrostatic interactions / Steric interactions**	**Distance (Å)**
**Co-crystallized (**OH-CH_2-pterin-pyrophosphate**)**	-86.62	ALA 199 :A, ARG 32 :A, ARG 52 :A, ARG 66 :A, ARG 87 :A, ARG 135 :A, ARG 176 :A, ARG 202 :A, ARG 219 :A, ARG 239 :A, ASN 11 :A, ASN 103 :A, ASP 42 :A, ASP 45 :A, ASP 84 :A, ASP 100 :A, ASP 104 :A, ASP 167 :A, ASP 213 :A, ASP 220 :A, GLN 105 :A, GLU 39:A, GLU 56:A, GLU 62:A,	O sp^2 (O8) - N sp^3 from LYS 303:A<br>N sp^2 (N1) - N sp^3 from LYS 303:A<br>N sp^2 (N1) - N sp^2 from ARG 239:A<br>N sp^2 (N7) - O sp^2 from ASP 167:A<br>N sp^2 (N6) - O sp^2 from ASP 167:A<br>N sp^2 (N6) - O sp^2 from ASN 103:A	2.397<br>2.944<br>3.535<br>2.326<br>2.826<br>2.415<br>2.926<br>3.584<br>2.846<br>3.350<br>3.457<br>2.947	**Electrostatic interactions**<br>O sp^2 (O2P) – Mn sp^3 269:A<br>O sp^2 (O4P) - Mn sp^3 269:A<br>O sp^2 (O5P) - Mn sp^3 269:A<br>O sp^2 (O4P) - N sp^2 from ARG 239:A<br>O sp^2 (O4P) - N sp^2 from ARG 239:A<br>O sp^2 (O5P) – N sp^2 from HIS 241:A<br>O sp^2 (O5P) - N sp^2 from HIS 241:A	3.563<br>4.461<br>4.257<br>3.603<br>2.846<br>4.477<br>2.755<br>4.336<br>4.246<br>3.457<br>3.724

		GLU 63:A, GLU 124:A, GLU 176:A, GLU 179:A, GLU 208:A, GLU 218:A, GLU 221:A, GLY 169 :A, HIS 55 :A, HIS 241 :A, ILE 9 :A, ILE 170 :A, LEU 197 :A, LYS 3:A, LYS 5:A, LYS 34:A, LYS 80:A, LYS 96:A, LYS 163:A, LYS 174:A, LYS 203:A, LYS 207:A, LYS 233:A, LYS 248:A, LYS 251:A, LYS 257 :A, MET 128 :A, PHE 172 :A, SER 50 :A, VAL 49 :A, VAL 126 :A, LYS 248 :B, LYS 251 :B.	N sp^2 (N5) - O sp^2 from ASN 103:A<br>N sp^2 (N4) - N sp^2 from GLN 105:A<br>O sp^2 (O4P) - N sp^2 from ARG 239:A<br>O sp^2 (O5P) - N sp^2 from ASN 11:A<br>O sp^2 (O6P) - N sp^2 from ARG 239:A<br>O sp^2 (O1P) - N sp^2 from ARG 52:A		O sp^2 (O6P) - N sp^2 from HIS 241:A<br>O sp^2 (O6P) - N sp^2 from ARG 239:A<br>O sp^2 (O6P) - N sp^2 from ARG 239:A<br>O sp^2 (O1P) - N sp^2 from ARG 52:A	
					**Steric interactions**<br>O sp^2(O8) - C sp^3 from ALA 199:A<br>C sp^2 (C8) - O sp^2 from ASP 167:A<br>N sp^2 (N6) - C sp^3 from ASN 103:A<br>N sp^2 (N4) - O sp^2 from ASP 84:A<br>O sp^2 (O4P) - O sp^2 from ASN 11:A<br>O sp^2 (O5P) - N sp^2 from HIS 241:A	3.168<br>2.903<br>3.157<br>2.621<br>3.163<br>2.755
**Compound 1a**	-89.15	ALA 199 :A, ARG 52 :A, ARG 202 :A, ARG 204 :A, ARG 219 :A, ARG 239 :A, ASN 103:A, ASP 45 :A, ASP 84 :A, ASP 167 :A, GLN 105 :A, HIS 241 :A, ILE 9 :A, ILE 170 :A, LEU 197 :A, LYS 3:A, LYS 203:A, LYS 207:A, MET 128 :A, PHE 172 :A, SER 201:A.	O sp^3 (O1) - N sp^2 from ARG 239:A<br>O sp^3 (O1) - N sp^2 from ARG 239:A<br>N sp^2 (N3) - N sp^2 from ARG 52:A<br>O sp^2 (O2) - N sp^2 from ARG 219:A<br>O sp^2 (O2) - N sp^2 from ARG 219:A	3.130<br>3.108<br>2.873<br>2.739<br>3.253	N sp^3 (N1) - N sp^2 from ARG 219:A<br>N sp^3 (N1) - N sp^2 from LYS 207:A<br>C sp^2 (C6) - N sp^2 from ARG 52:A<br>C sp^2 (C8) - N sp^2 from ARG 239:A<br>C sp^2 (C11) - O sp^2 from ASP 84:A<br>C sp^2 (C10) - S sp^3 from MET 128:A	3.107<br>3.032<br>3.170<br>3.096<br>2.709<br>2.893

**Compound 1b**	-53.94	ARG 52 :A, ARG 202 :A, ARG 204 :A, ARG 219 :A, ARG 239 :A, ASN 11:A, ASP 84 :A, GLN 105 :A, HIS 241 :A, LYS 203:A, PHE 172:A, SER 50 :A, SER 201:A, VAL 49 :A.	N sp^3 (N1) - O sp^2 from ASP 84:A<br>O sp^2 (O2) - N sp^2 from ARG 219:A<br>O sp^2 (O3) - N sp^2 from GLN 105:A<br>N sp^2 (N3) - N sp^2 from ARG 52:A<br>N sp^2 (N3) - N sp^2 from ARG 52:A	2.993<br>2.505<br>2.586<br>2.841<br>3.192	N sp^3 (N1) - C sp^2 from ARG 239:A<br>N sp^3 (N1) - N sp^2 from ARG 239:A<br>N sp^3 (N1) - N sp^2 from ARG 239:A<br>C sp^2 (C20) - N sp^2 from ARG 52:A	3.144<br>3.128<br>2.965<br>2.821
**Compound 1c**	-49.92	ARG 52 :A, ARG 202 :A, ARG 204 :A, ARG 219 :A, ARG 239 :A, ASN 11:A, ASP 84 :A, HIS 241 :A, ILE 9 :A, LYS 203:A, LYS 207:A, PHE 172:A, PRO 216 :A, SER 201:A.	O sp^2 (O2) - N sp^2 from ARG 239:A	2.902	N sp^3 (N1) - N sp^2 from ARG 239:A<br>N sp^3 (N1) - N sp^2 from ARG 239:A	2.656<br>3.182
**Compound 1d**	-65.81	ARG 52 :A, ARG 202 :A, ARG 204 :A, ARG 219 :A, ARG 239 :A, ASN 11:A, HIS 241 :A, ILE 9 :A, LYS 203:A, LYS 207:A, SER 201:A.	N sp^3 (N1) - N sp^2 from HIS 241:A<br>O sp^2 (O3) - N sp^2 from ARG 239:A<br>O sp^2 (O3) - N sp^2 from ARG 239:A	2.967<br>3.159<br>2.594	N sp^3 (N1) - N sp^2 ASN 11:A<br>Cl sp^3 (Cl 1) - C sp^3 from LYS 207:A	3.113<br>2.874
**Compound 1e**	-60.08	ARG 52 :A, ARG 202 :A, ARG 204 :A, ARG 219 :A, ARG 239 :A, ASP 84 :A, GLN 105 :A, LYS 203:A, LYS 207:A, PHE 172 :A, PRO 216 :A, SER 50 :A, SER 201:A, VAL 49 :A.	O sp^2 (O2) - N sp^2 from ARG 239:A<br>O sp^2 (O3) - N sp^2 from GLN 105:A	3.027<br>3.121	N sp^3 (N1) - N sp^2 from ARG 239:A	2.771

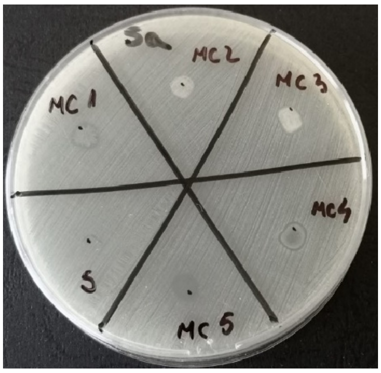

(a)

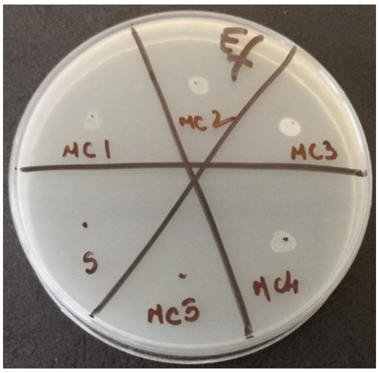

(b)

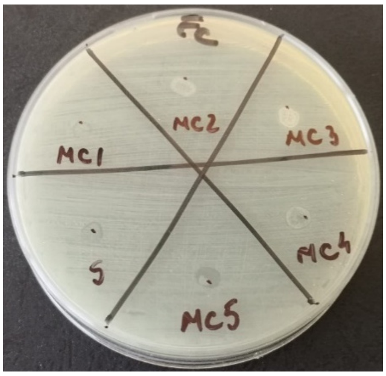

(c)

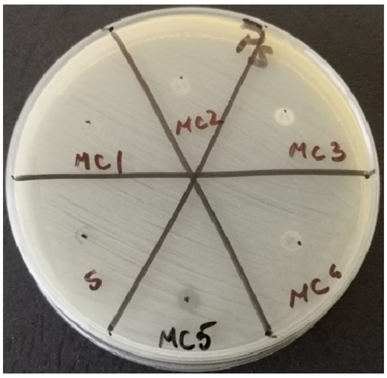

(d)

Figure S30. Qualitative evaluation of the antimicrobial activity of Schiff bases ***1a–e****: (a) Staphylococcus aureus ATCC 25923; (b) Enterococcus faecalis ATCC 29212; (c) Escherichia coli ATCC 25922; (d) Pseudomonas aeruginosa ATCC 27853. Corresponding notations:* ***1a*** *(MC2),* ***1b*** *(MC5),* ***1c*** *(MC1),* ***1d*** *(MC4),* ***1e*** *(MC3), S (Sulphanilamide).*

Table S5. Presentation of minimum inhibitory concentrations for antibacterial activity and bacterial adherence assay for Schiff bases 1a-e. Corresponding notations: SA: Staphylococcus aureus ATCC 25923; EF: Enterococcus faecalis ATCC 29212; EC: Escherichia coli ATCC 25922; PA: Pseudomonas aeruginosa ATCC 27853.

**Compounds**	**Minimum inhibitory concentration (mg/mL) (mean ± SD)**							
	**Antibacterial activity**				**Bacterial adherence to inert substrata**			
	**SA**	**EF**	**EC**	**PA**	**SA**	**EF**	**EC**	**PA**
**1a**	0.059 ± 0.028	0.312	0.625	0.469 ± 0.221	0.078	0.625	0.625	0.625
**1b**	0.014 ± 0.007	0.938 ± 0.442	0.625	0.234 ± 0.110	0.029 ± 0.014	1.25	0.625	0.312
**1c**	0.029 ± 0.014	0.469 ± 0.221	0.625	0.469 ± 0.221	0.059± 0.028	0.625	0.625	0.625
**1d**	0.019	0.312	0.625	0.312	0.039	0.625	0.625	0.312
**1e**	0.039	0.156	0.625	0.625	0.117± 0.055	0.234 ± 0.110	0.625	0.625
**Sulfanilamide**	0.039	1.25	0.625	0.156	0.078	0.938 ± 0.442	0.625	0.234 ± 0.110

*Table S6. Presentation of the lowest doses to induce a statistically significant response in terms of HT-29 and LN229 cells viability decrease after 24h and 48h of treatment with Schiff base **1a-e**.*

Cell line	Compounds	Time	First dose to induce significant cytotoxic effect
HT-29 colorectal adenocarcinoma cells	Sulfanilamide	24h	200 µg/ml (****, p<0.0001)
		48h	5 µg/ml (****, p<0.0001)
	1a	24h	40 µg/ml (**, p<0.01)
		48h	5 µg/ml (****, p<0.0001)
	1b	24h	40 µg/ml (****, p<0.0001)
		48h	40 µg/ml (**, p<0.01)
	1c	24h	40 µg/ml (****, p<0.0001)
		48h	5 µg/ml (****, p<0.0001)
	1d	**24h**	**5 µg/ml (****, p<0.0001)**
		48h	1.6 µg/ml (****, p<0.0001)
	1e	24h	8 µg/ml (**, p<0.01)
		48h	1.6 µg/ml (****, p<0.0001)
LN229 glioblastoma cells	Sulfanilamide	24h	40 µg/ml (**, p<0.01)
		48h	5 µg/ml (***, p<0.001)
	1a	24h	5 µg/ml (**, p<0.01)
		48h	8 µg/ml (**, p<0.01)
	1b	24h	5 µg/ml (*, p<0.05)
		48h	1.6 µg/ml (****, p<0.0001)
	1c	**24h**	**5 µg/ml (****, p<0.0001)**
		48h	1.6 µg/ml (****, p<0.0001)
	1d	24h	8 µg/ml (**, p<0.01)
		48h	1.6 µg/ml (****, p<0.0001)
	1e	24h	8 µg/ml (*, p<0.05)
		48h	1.6 µg/ml (****, p<0.0001)
